# PAXX binding to the NHEJ machinery explains functional redundancy with XLF

**DOI:** 10.1126/sciadv.adg2834

**Published:** 2023-05-31

**Authors:** Murielle Seif-El-Dahan, Antonia Kefala-Stavridi, Philippe Frit, Steven W. Hardwick, Dima Y. Chirgadze, Taiana Maia De Oliviera, Jessica Andreani, Sébastien Britton, Nadia Barboule, Madeleine Bossaert, Arun Prasad Pandurangan, Katheryn Meek, Tom L. Blundell, Virginie Ropars, Patrick Calsou, Jean-Baptiste Charbonnier, Amanda K. Chaplin

**Affiliations:** ^1^ Institute for Integrative Biology of the Cell (I2BC), Institute Joliot, CEA, CNRS, Université Paris-Saclay, 91198 Gif-sur-Yvette cedex, France.; ^2^ Department of Biochemistry, University of Cambridge, Sanger Building, Tennis Court Road, Cambridge CB2 1GA, UK.; ^3^ Institut de Pharmacologie et Biologie Structurale, IPBS, Université de Toulouse, CNRS, UPS, Toulouse, France.; ^4^ Cryo-EM Facility, Department of Biochemistry, University of Cambridge, Sanger Building, Tennis Court Road, Cambridge CB2 1GA, UK.; ^5^ AstraZeneca R&D, Discovery Sciences, Mechanistic and Structural Biology, Cambridge, UK.; ^6^ Leicester Institute for Structural and Chemical Biology, Department of Molecular and Cell Biology, University of Leicester, Leicester, UK.; ^7^ College of Veterinary Medicine, Department of Microbiology & Molecular Genetics, Department of Pathobiology and Diagnostic Investigation, Michigan State University, East Lansing, MI 48824, USA.

## Abstract

Nonhomologous end joining is a critical mechanism that repairs DNA double-strand breaks in human cells. In this work, we address the structural and functional role of the accessory protein PAXX [paralog of x-ray repair cross-complementing protein 4 (XRCC4) and XRCC4-like factor (XLF)] in this mechanism. Here, we report high-resolution cryo–electron microscopy (cryo-EM) and x-ray crystallography structures of the PAXX C-terminal Ku-binding motif bound to Ku70/80 and cryo-EM structures of PAXX bound to two alternate DNA-dependent protein kinase (DNA-PK) end-bridging dimers, mediated by either Ku80 or XLF. We identify residues critical for the Ku70/PAXX interaction in vitro and in cells. We demonstrate that PAXX and XLF can bind simultaneously to the Ku heterodimer and act as structural bridges in alternate forms of DNA-PK dimers. Last, we show that engagement of both proteins provides a complementary advantage for DNA end synapsis and end joining in cells.

## Teaser

PAXX and XLF structurally bridge alternate forms of DNA-PK dimers, explaining the redundancy between XLF and PAXX in NHEJ.

## INTRODUCTION

Nonhomologous end joining (NHEJ) is dependent on several canonical proteins, namely, the heterodimer Ku70/80 (Ku), the large DNA-dependent protein kinase catalytic subunit (DNA-PKcs), DNA ligase IV (Lig4), x-ray repair cross-complementing protein 4 (XRCC4), and XRCC4-like factor (XLF) (*1*, *2*). These core proteins are sufficient for ligation of double-strand breaks (DSBs); however, several accessory proteins with overlapping and redundant functions are essential for efficient DSB repair under specific conditions (*3*).

Following a DSB, the first NHEJ protein that binds the break site is Ku, where it rapidly recruits DNA-PKcs to form the DNA-PK complex or holoenzyme (*4*, *5*). Recently, using cryo–electron microscopy (cryo-EM), we revealed that DNA-PK can exist as a dimer mediated by the C terminus of Ku80 allowing bridging of broken DNA ends (*6*). Furthermore, addition of the core proteins Lig4, XRCC4, and XLF led to the discovery of an alternate DNA-PK dimer mediated by XLF (*7*, *8*). The two DNA-PK dimers represent alternate forms of the long-range (LR) synaptic complexes that can transition to a short-range assembly following the removal of DNA-PKcs (*8*, *9*).

XLF contains a Ku-binding motif (KBM) at its C terminus, and this short, conserved region has been shown to interact directly with the Ku80 von Willebrand type A (vWA) domain, inducing an outward rotation of this region (*10*). PAXX (paralog of XRCC4 and XLF) is a more recently identified NHEJ accessory protein, which structurally resembles XRCC4 and XLF [reviewed in (*11*)]. The C terminus of PAXX also contains a KBM denoted P-KBM (*12*–*14*). In contrast to the interaction of XLF with Ku80, PAXX has been shown to interact directly with Ku70 (*14*). Single-molecule studies indicate that PAXX facilitates and stabilizes synapsis of DNA ends during NHEJ (*15*). PAXX deficiency leads to no or mild sensitivity to DNA damage (*13*, *14*, *16*–*24*). PAXX has been shown to be functionally redundant with XLF (*18*–*20*, *25*, *26*), and deletion of both genes is synthetically lethal in mice (*21*, *22*, *24*). However, the protein levels of PAXX and XLF have been shown to vary inversely in certain cancer tissues, suggesting that these two proteins may have more specific independent functional roles (*27*).

To address the role of PAXX in DNA synapsis and to understand how it functionally overlaps with XLF, we have structurally characterized the specific binding of PAXX to Ku and larger NHEJ assemblies. We show that mutations on specific residues within either Ku70 or PAXX encompassing the interaction site prevent recruitment of PAXX at DSB sites. We also present cryo-EM structures of PAXX bound via Ku70 to the two LR DNA-PK dimer forms. We demonstrate that PAXX and XLF can bind simultaneously to Ku and that PAXX bridges the Ku80-mediated dimer of DNA-PK but cannot structurally substitute for XLF in the XLF-mediated DNA-PK dimer. We also show that PAXX loss in cells exacerbates the impact of XLF deficiency on DNA end synapsis and joining. The stabilization of alternate LR dimeric assemblies explains the functional redundancy of PAXX and XLF.

## RESULTS AND DISCUSSION

### Visualization of Ku70 binding to the KBM of PAXX

**Fig. 1.**
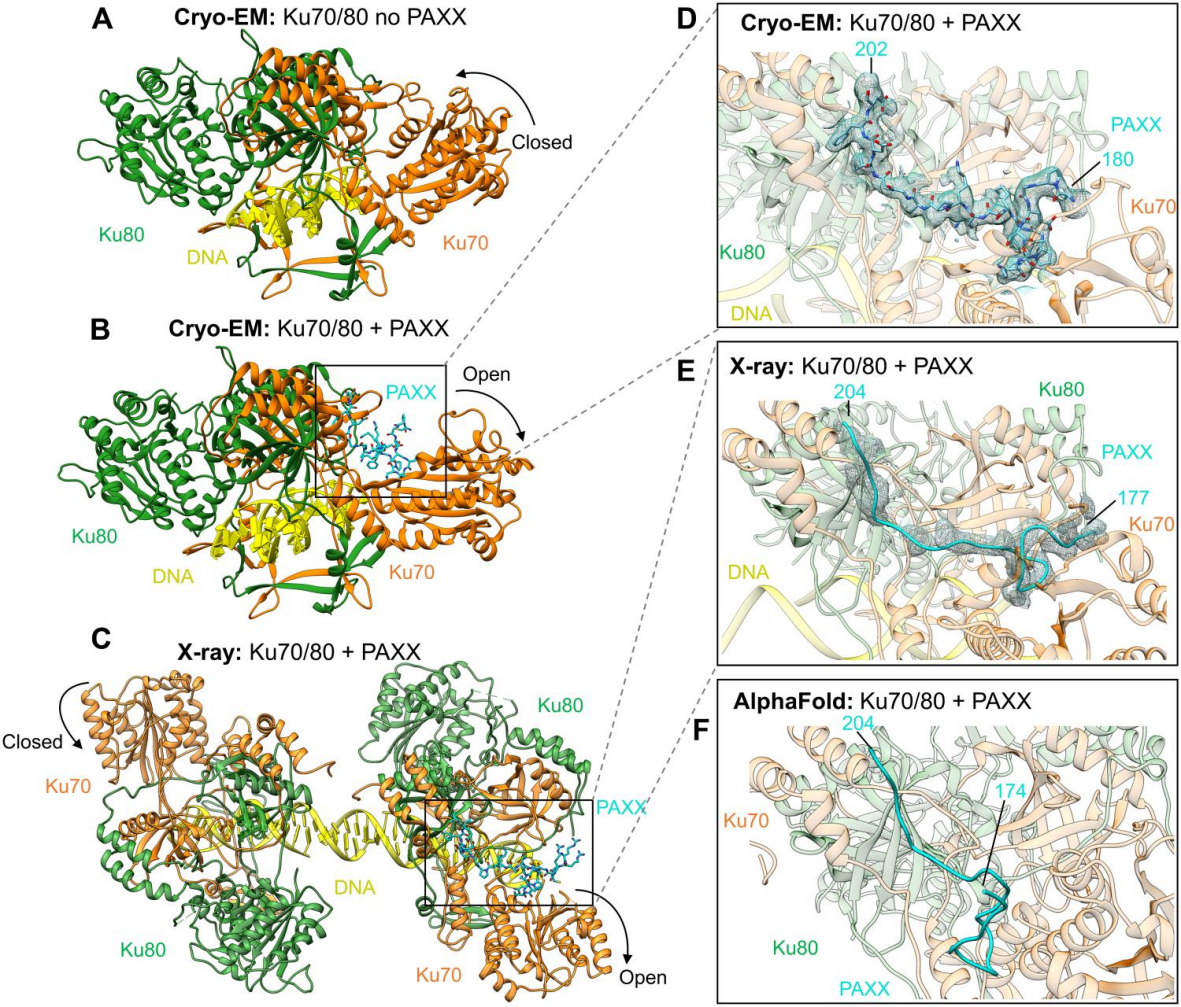
Structures of the PAXX KBM bound to Ku70/80. (**A**) Cryo-EM structure of Ku70/80-DNA with no PAXX bound to 2.7-Å resolution (PDB: 7ZVT). (**B**) Cryo-EM structure of Ku70/80-DNA with the P-KBM bound to 2.8-Å resolution. (**C**) X-ray crystallography structure of Ku70/80, with the P-KBM bound to one of the heterodimers in the crystallographic unit to 2.97-Å resolution. (**D**) Enlarged view of the P-KBM bound to Ku70/80 in the cryo-EM structure with the density for the peptide shown as mesh. (**E**) Enlarged view of the P-KBM bound to Ku70/80 in the x-ray structure with the density shown as a mesh. (**F**) AF model of the P-KBM bound to Ku70/80. Ku70 is shown in orange, Ku80 in green, DNA in yellow, and PAXX in cyan.

To define the molecular details of the interaction between PAXX and Ku, we pursued several strategies in parallel, including cryo-EM, x-ray crystallography, and computational modeling with AlphaFold-Multimer (AF). We collected cryo-EM data of Ku bound to DNA and PAXX. We obtained a cryo-EM map representing the Ku DNA complex bound to PAXX at 2.8-Å resolution (Fig. 1, A and B, table S1, and figs. S1 and S2). Within the cryo-EM map containing PAXX, we could confidently build residues 180 to 202 of the P-KBM (Fig. 1D). Although full-length PAXX was added to the cryo-EM sample, the only density observed was that of the P-KBM, in agreement with previous studies suggesting that the P-KBM is critical for the interaction with Ku [reviewed in (*11*, *12*)]. We observe an opening in the vWA domain of Ku70 upon PAXX binding, which is larger than the opening observed when Ku binds to DNA-PKcs to form the DNA-PK holoenzyme (fig. S3A).

In addition, we obtained crystals of Ku_ΔC_ (a version of the Ku70/80 heterodimer with truncations in the C termini of both proteins; fig. S4A), in complex with a peptide corresponding to the P-KBM of PAXX. The crystal structure was determined by molecular replacement to 2.97-Å resolution, and the x-ray map shows the presence of two Ku_ΔC_ molecules in the asymmetric unit bound to DNA. The two DNA molecules bound to the Ku_ΔC_ interact through their single-strand overhang, positioning the two Ku heterodimers at close proximity without direct protein:protein contact (Fig. 1C and table S2). Although two Ku molecules are present in the crystallographic asymmetric unit, only one is complexed with the P-KBM, and the entire 28 residues of this peptide were built into this additional electron density (residues 177 to 204) (Fig. 1E). In both the cryo-EM and x-ray models, it is apparent that the P-KBM of PAXX binds within a cleft on the surface of Ku associated with a large outward rotation of the Ku70 vWA domain, when comparing to the structure of Ku in the absence of PAXX [Protein Data Bank (PDB): 7ZVT, Electron Microscopy Data Bank (EMDB): 14986] (Fig. 1, fig. S4, and movie S1). An overlay of the “open” PAXX-bound cryo-EM and x-ray structure and an overlay of the “closed” no PAXX-bound structures illustrate that, between the two techniques, the movement in the vWA of Ku70 is near identical (with only a slight closing in the x-ray compared to the cryo-EM; fig. S3). We can therefore highlight that the crystal lattices do not influence the opening and closing of Ku70.

Last, we also used AF to predict the interaction between Ku and PAXX and to compare with our experimental structures (Fig. 1F and fig. S4). The program succeeded in positioning the last eight residues of the P-KBM in a similar location to that observed in our structures (Fig. 1F) but did not predict an opening of Ku70 vWA. Therefore, residues within the Ku70 binding pocket, in contact with the N-terminal part of P-KBM as seen in the experimental structures, were not successfully modeled. We subsequently performed AF analysis using only the vWA of Ku70 and the P-KBM. In this reduced system, AF succeeds in more accurately predicting the position of the P-KBM bound to the Ku70 vWA (fig. S4).

These structural data therefore establish that PAXX interacts via its C-terminal P-KBM motif with the vWA domain of Ku70. From the cryo-EM data, we observed no evidence for a direct interaction between the head and coiled-coil domains of PAXX (residues 1 to 144) and Ku, in agreement with the previous study that reports a similar affinity of PAXX or P-KBM for the Ku/DNA complex (*14*).

### Molecular basis of the P-KBM interaction with Ku70

**Fig. 2.**
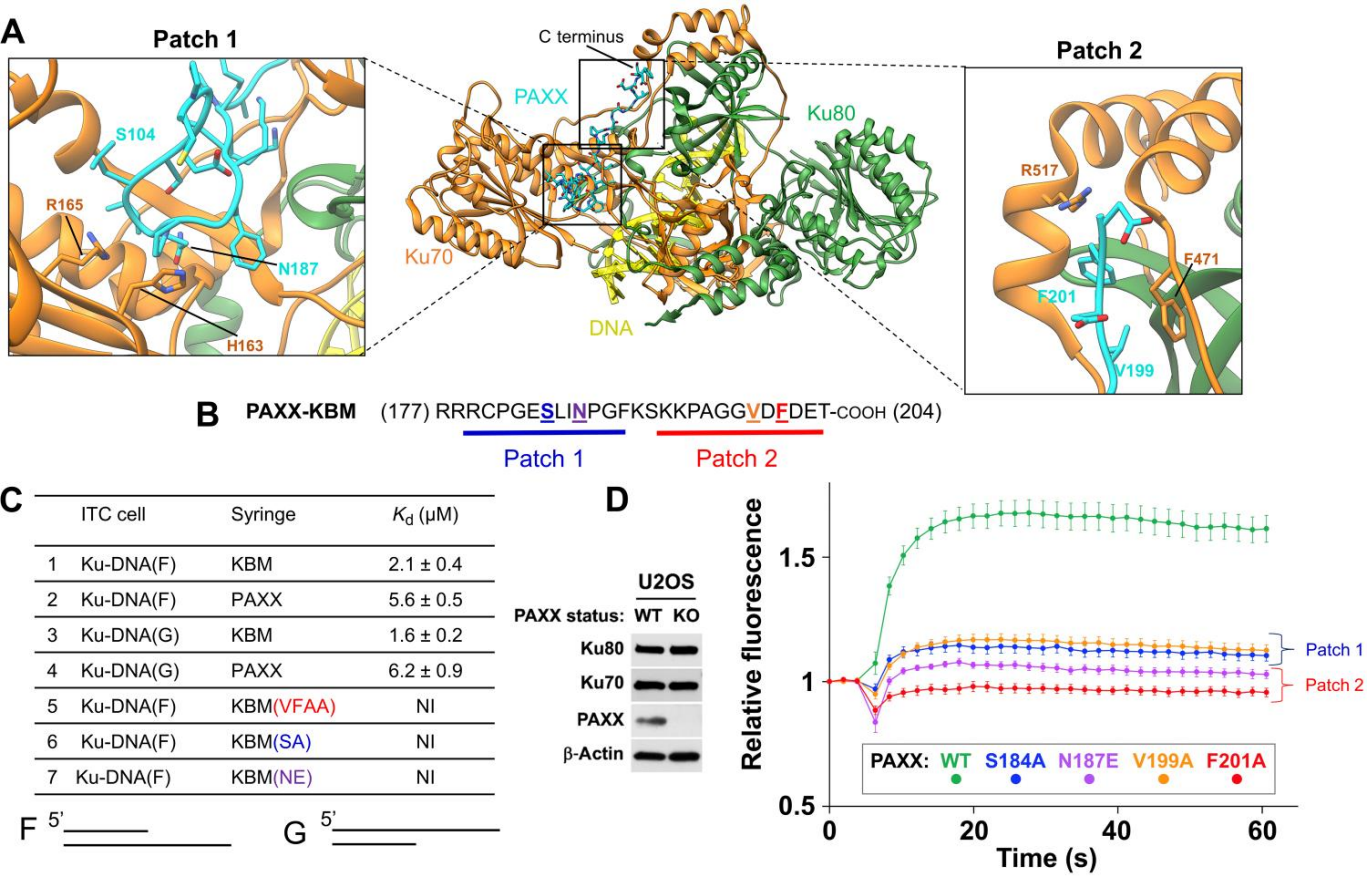
Mutagenesis of residues within the P-KBM of PAXX affects Ku binding and recruitment to DNA damage sites. (**A**) The cryo-EM structure of Ku70/80-DNA bound to the P-KBM of PAXX, with interaction of Patches 1 and 2 illustrated, and insets show them enlarged with residues labeled. Ku70 is in orange, Ku80 is in green, DNA is in yellow, and PAXX is in cyan. (**B**) The sequence of the C terminus of PAXX, with interaction of Patches 1 and 2 identified and residues mutated colored, in bold and underlined. (**C**) Isothermal titration calorimetry (ITC) data table for Ku70/80-DNA (either F or G DNA shown below the table) with PAXX (full-length, KBM, or mutants), *K*_d_ values are indicated in millimolars. NI: no interaction. (**D**) Laser microirradiation experiment monitoring the recruitment of WT PAXX or mutants to sites of DNA damage in U2OS PAXX knockout (KO) cells. Results were plotted as mean values ± SEM. Ku70 mutants of the P-KBM of PAXX are colored accordingly. Left: Western blot confirming PAXX KO in U2OS cells.

The P-KBM is engaged in a groove located between the vWA of Ku70, the core of Ku, and the C-terminal arm of Ku70 (Fig. 1). This interaction is bipartite, with two clear sites of interaction on Ku. Patch 1 is formed between the N-terminal part of the P-KBM and a groove formed between the vWA domain of Ku70 and the core of Ku. Patch 2 is mediated by the C terminus of the P-KBM and two helices of Ku70 packed against the Ku core (Fig. 2, A and B). Multiple sequence alignment of PAXX and Ku from various organisms highlights several conserved residues in the interaction sites (figs. S4B and S5).

From the sequence and structural analysis, we designed mutations to disrupt either Patch 1 or Patch 2. For Patch 1, S184A and N187E mutations were introduced in PAXX, and H163A and R165E were introduced in Ku70. For Patch 2, we introduced two PAXX mutations (V199A and F201A) alone or as a double mutant (V199A + F201A, herein termed VFAA) and two Ku70 mutations (F471E and R517E) (Fig. 2, A and B). The effects of these mutations were assessed both in vitro and in cells as described below.

### Impact of mutations in the P-KBM

We analyzed by isothermal titration calorimetry (ITC) the affinity of Ku for constructs containing the wild-type (WT) or mutant versions of the P-KBM. In our experimental setup, we used Ku in complex with either oligonucleotide F [15 base pairs (bp) with a 15–nucleotide (nt) overhang in 5′] or with oligonucleotide G (15 bp with a 15-nt overhang in 3′) as used previously (*14*). Initially, we compared the affinity of Ku in complex with the two DNA oligonucleotides for either WT P-KBM peptide or full-length PAXX protein. In this experiment, we measured similar affinities in the micromolar range, confirming that the P-KBM drives the interaction between PAXX and Ku (Fig. 2C). Under the same conditions, the three P-KBM mutant peptides (VFAA on Patch 1 and S184A and N187E on Patch 2) showed no interaction with Ku (Fig. 2C and fig. S6). In cells, PAXX is recruited to laser-induced DNA damage sites via the interaction of its C-terminal KBM with Ku (*20*). We performed similar nuclear microirradiation experiments to monitor the recruitment of variants of PAXX to sites of DNA damage. Within PAXX knockout (KO) U2OS cells, we observed that all mutations tested within the P-KBM markedly reduced the recruitment of green fluorescent protein (GFP)–PAXX to DNA damage sites (Fig. 2D). These data establish the importance of these residues for the interaction with Ku as predicted from our structural analysis. These data are also in agreement with previous data reporting functional defects of the VFAA P-KBM mutant (*13*).

### Impact of mutations on Ku70

**Fig. 3.**
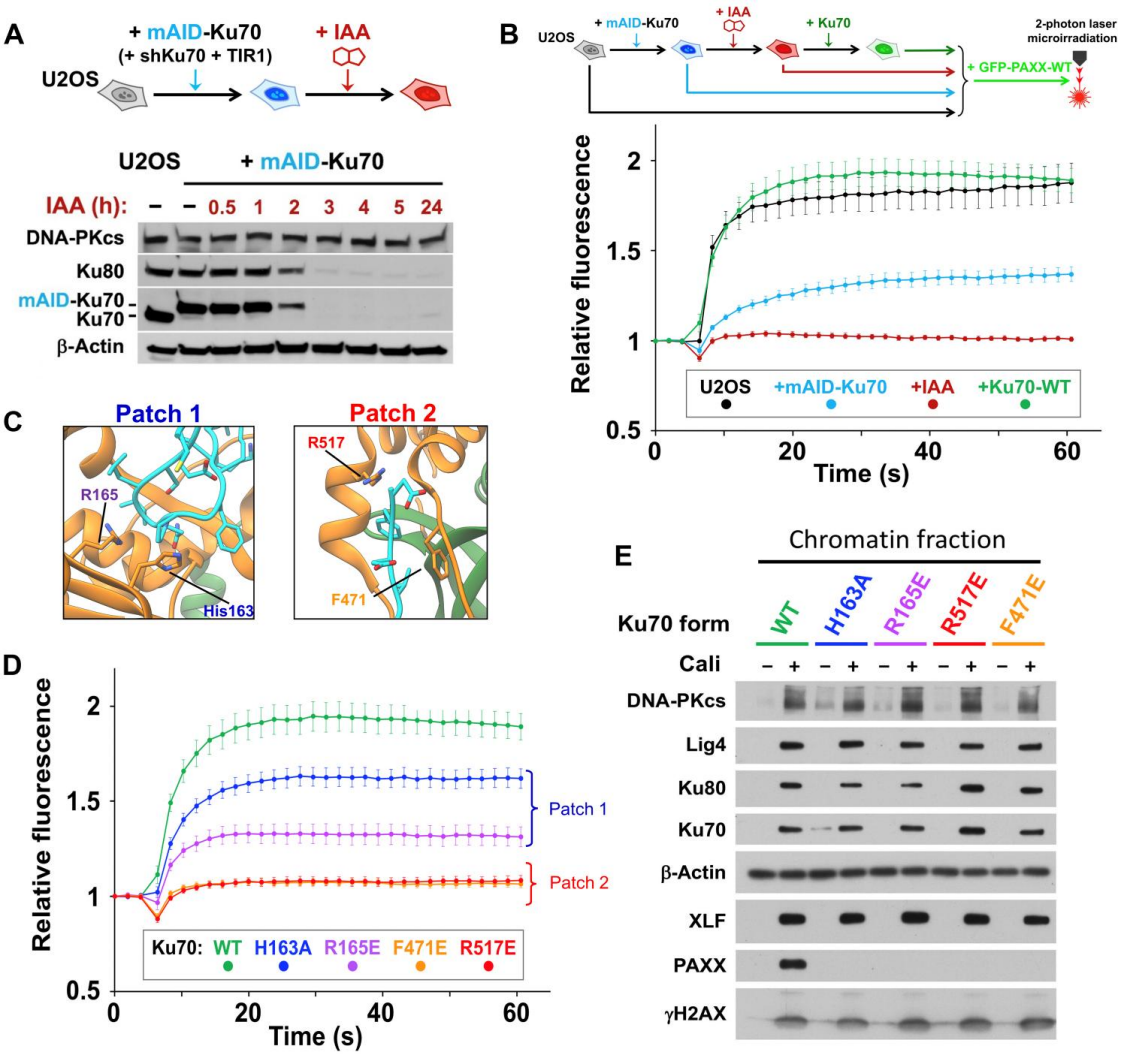
Mutagenesis of PAXX-binding sites in Ku70 impairs PAXX recruitment at DSBs using a degron approach. (**A**) Depiction of the system used for Ku degradation upon auxin (IAA) addition in U2OS cells. (**B**) Principle of the microirradiation experiment (top) and quantifications (bottom) of GFP-PAXX accumulation at laser-induced DNA damage in control U2OS cells or U2OS cells expressing mAID-Ku70 without or with WT-Ku70 complementation, under conditions without or with IAA. Results were plotted as mean values ± SEM. (**C**) Structural maps of mutated residues in the Ku70 interface with PAXX. (**D**) Quantification of GFP-PAXX accumulation at laser-induced DNA damage in U2OS cells expressing WT or mutated Ku70 as indicated. Results were plotted as mean values ± SEM. Ku70 mutants are colored accordingly. (**E**) Western blot of chromatin fractions from U2OS cells expressing WT or mutated Ku70 as indicated, treated or not with 3 nM calicheamicin (Cali) for 1 hour.

We also purified three mutants of Ku70 complexed to WT Ku80 (fig. S7). All these mutants have a similar thermal stability compared to Ku WT but show no residual interaction with the P-KBM as assessed by ITC (fig. S7). To assess the impact of Ku70 mutations in cells, we engineered a system in U2OS cells to allow replacement of endogenous Ku70 by mutant forms of the protein (Fig. 3A). As Ku is essential for the viability of human cells, we first knocked down endogenous Ku70 expression via the constitutive expression of a short hairpin–mediated RNA (shRNA) combined with cell rescue through expression of Ku70 tagged with a mini auxin-inducible degron (mAID). This allows for rapid degradation of endogenous Ku70 upon addition of auxin [indole-3-acetic acid (IAA)] (*28*). This system permits a strong and fast Ku70 depletion, with concomitant Ku80 depletion due to reciprocal stabilization of both Ku subunits (Fig. 3A). We found that PAXX recruitment to damage sites was fully Ku dependent because it was abolished under Ku depletion conditions (Fig. 3B, red line). PAXX recruitment to microirradiated areas was restored to levels similar to those in native U2OS cells by expression of recombinant WT Ku70 (Fig. 3B, green line).

Having established a methodology for assessing PAXX recruitment to DNA damage sites, we then replaced endogenous Ku70 with mutated forms on either Patch 1 or Patch 2 (Fig. 3C and fig. S8). Although Ku mobilization to damaged sites was similar for WT and mutant forms of Ku70 (fig. S8), all of Ku70 mutants tested in both Patch 1 and Patch 2 impaired GFP-PAXX accrual at laser-induced DNA damage sites to various extents, with mutations in Patch 2 being the most detrimental (Fig. 3D).

To challenge these data with an orthogonal method, we performed cell fractionation following treatment with the DNA damaging agent calicheamicin, allowing the detection of Ku-dependent stable recruitment of NHEJ proteins to the chromatin fraction containing the DSB marker γH2AX, as previously reported (*29*) (fig. S8, C and D). Ku70 mutants were expressed at similar levels to WT, and the mutations did not impact on the expression of other NHEJ proteins, including PAXX (fig. S8E). However, PAXX was not detected in damaged chromatin fraction when either Patch 1 or Patch 2 of Ku70 was mutated, while, in contrast, all the other NHEJ proteins tested including XLF were recruited at levels comparable to the control cells expressing WT Ku70 (Fig. 3E). Together, these findings validate our structural data and demonstrate that the vWA domain of Ku70 is crucial for the interaction with PAXX in cells.

### PAXX bridges the Ku80-mediated DNA-PK dimer

**Fig. 4.**
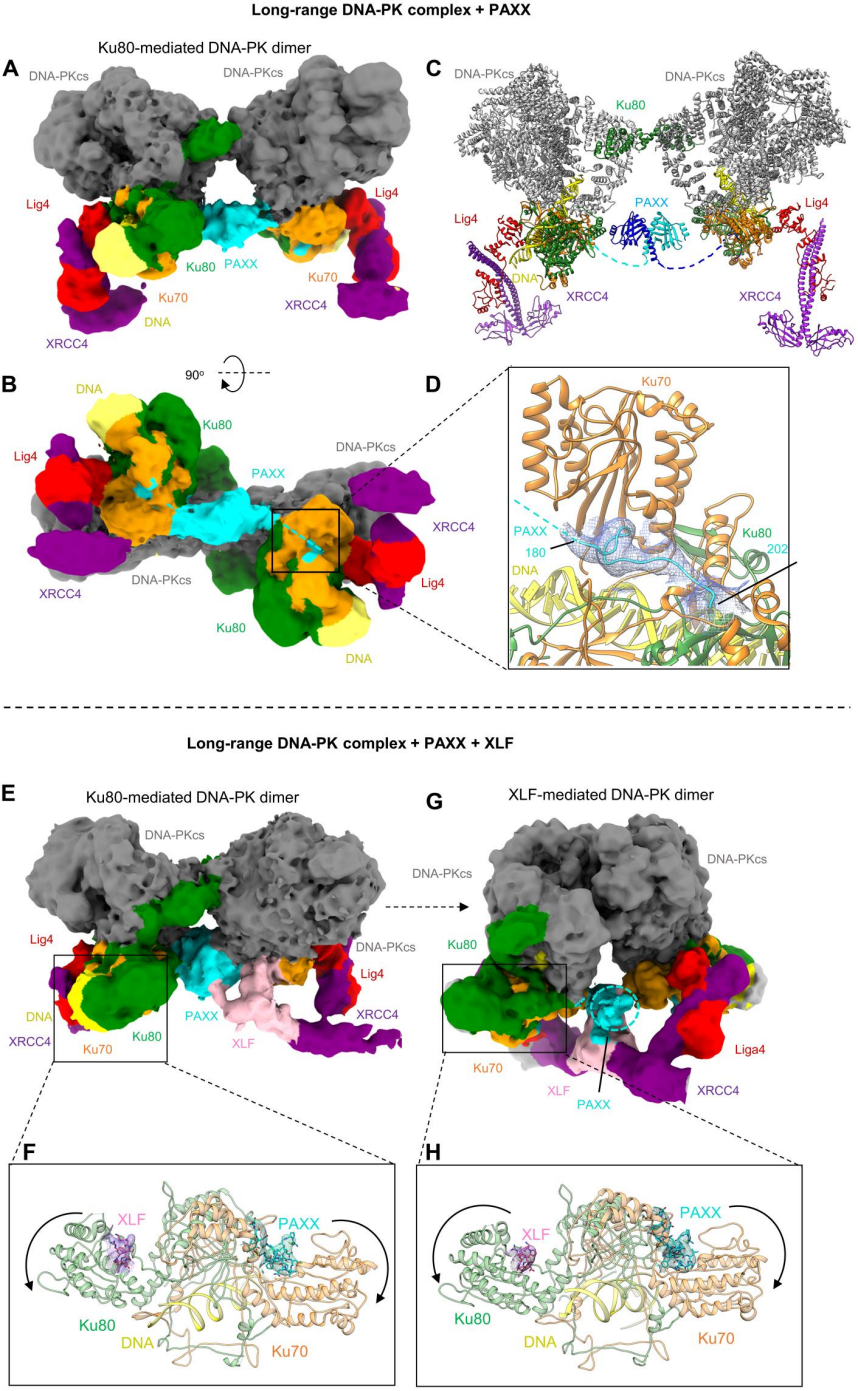
Cryo-EM structures of DNA-PK LR synaptic complexes with PAXX. (**A** and **B**) Two views of the DNA-PK Ku80-mediated LR cryo-EM map with PAXX. DNA-PKcs is shown in gray, Ku70 in orange, Ku80 in green, DNA in yellow, XRCC4 in purple, Lig4 in red, and PAXX in cyan. (**C**) Structure of the DNA-PK LR Ku80-mediated dimer with PAXX. Each half of the PAXX homodimer is colored different shades of blue, and the dashed lines indicate where the C-terminal tail links to the P-KBM within Ku70. (**D**) Close up view of the P-KBM of PAXX bound to Ku70 in the LR DNA-PK Ku80-mediated dimer, density is shown as a mesh. (**E**) Cryo-EM map of the Ku80-mediated DNA-PK LR synaptic dimer with PAXX (cyan) and XLF (pink). (**F**) Enlarged view of Ku70/80 from the LR Ku80-mediated DNA-PK complex, with the peptide of XLF shown as pink sticks and the density as pink mesh and the peptide of PAXX in cyan sticks and cyan mesh. (**G**) Cryo-EM map of the XLF-mediated DNA-PK LR synaptic dimer with PAXX and XLF bound. (**H**) Enlarged view of Ku70/80 from the LR Ku80-mediated DNA-PK complex, with the same color codes for the XLF and PAXX peptides than in (F).

Having extensively characterized in vitro and in cells the interaction between the P-KBM and Ku, we next addressed the role of PAXX binding within the DNA-PK holoenzyme. We collected cryo-EM data of a complex of DNA-PK, Lig4, XRCC4, and PAXX. We identified DNA-PK dimers mediated by the C terminus of Ku80, as described previously (*6*), and generated a cryo-EM map of this DNA-PK dimer to 4.6-Å overall resolution (Fig. 4, A to D, and figs. S9 and S10). The two protomers of DNA-PK were then locally refined to resolutions of 3.8 and 3.9 Å and combined into a composite map for presentation purposes. Within this dimer, density corresponding to the C-terminal P-KBM of PAXX is observed, engaged within the vWA of Ku70 in an open conformation in agreement with our studies using Ku in isolation (Fig. 4, B and D). In addition, we observe an area of extra density compared to the Ku80-mediated dimer alone (PDB: 6ZHE) (fig. S11). This density is located in the center of the DNA-PK dimer and connects to the density corresponding to the P-KBM engaged with Ku70 on both DNA-PK protomers. We therefore conclude that the density central to the DNA-PK dimer corresponds to the head and coiled-coil domains of PAXX (Fig. 4, A to C).

Although the density is of low resolution in this region, we were able to dock the head and coiled-coil domains of PAXX using the ADM_EM program (*30*) and perform a full exhaustive rigid body search to optimally fit the PAXX homodimer. Modeling of PAXX in this position suggests a domain swap like interaction; as the stalks of the PAXX homodimer separate toward the C terminus, this favors docking of the P-KBM on the opposite DNA-PK protomer to the head domain of PAXX (Fig. 4C and fig. S12).

### PAXX and XLF can bind to Ku70/80 simultaneously

In the absence of XLF, we did not observe any cryo-EM particles resembling the XLF-mediated DNA-PK dimer reported recently (*7*, *8*). This indicates that PAXX cannot structurally substitute for XLF to allow the formation of this dimeric form. However, to understand whether the binding of PAXX and XLF to DNA-PK is mutually exclusive, we prepared a cryo-EM sample of DNA-PK, Lig4, XRCC4, PAXX, and XLF. Within this dataset, we observed particles corresponding to both the DNA-PK dimer mediated by the C terminus of Ku80 and the DNA-PK dimer mediated by XLF, further confirming the necessity of XLF for formation of this alternate dimeric assembly (Fig. 4, E to H, and figs. S13 to S15). For the dimer mediated by Ku80, in addition to the central density corresponding to PAXX, we observed weak density corresponding to XLF bound to XRCC4, in an arrangement similar to the binding of XLF to the DNA-PK monomer as previously described (PDB:7NFE) (*7*) (Fig. 4E). Furthermore, we observed density corresponding to the P-KBM of PAXX bound to Ku70 and density for the X-KBM of XLF bound to Ku80 in agreement with the previous study (*10*) (Fig. 4F). The binding of both KBM’s results in an opening of both vWA domains of Ku70 and Ku80 of similar amplitude shows that both proteins can bind simultaneously within this dimer (Fig. 4, E and F). However, it is not clear from our data whether binding of PAXX to Ku aids the binding of XLF (or vice versa); however, the binding of one may provide structural stability and aid the binding of the second factor, which requires further investigation.

For the dimer mediated by XLF, we observed the density for XLF central within this dimeric assembly, as shown previously (*7*), and the additional weak density for either side of the XLF stalk that may correspond to the globular domain of PAXX (Fig. 4G). Similar to the Ku80-mediated dimer, when focusing on Ku, density is clearly visible for both the KBMs of XLF and PAXX, and both Ku70 and Ku80 vWA domains are in an open conformation (Fig. 4H). We conclude therefore that XLF and PAXX can also bind simultaneously to the XLF-mediated dimer of DNA-PK. It does indeed look as though PAXX and XLF can form direct interactions with each other in this structure; however, we did not observe a direct interaction when tested. It is, therefore, likely that they are only interacting when under the constraints of the DNA-PK LR complex with DNA-PKcs stabilizing the proteins and XRCC4 head interactions with XLF.

**Fig. 5.**
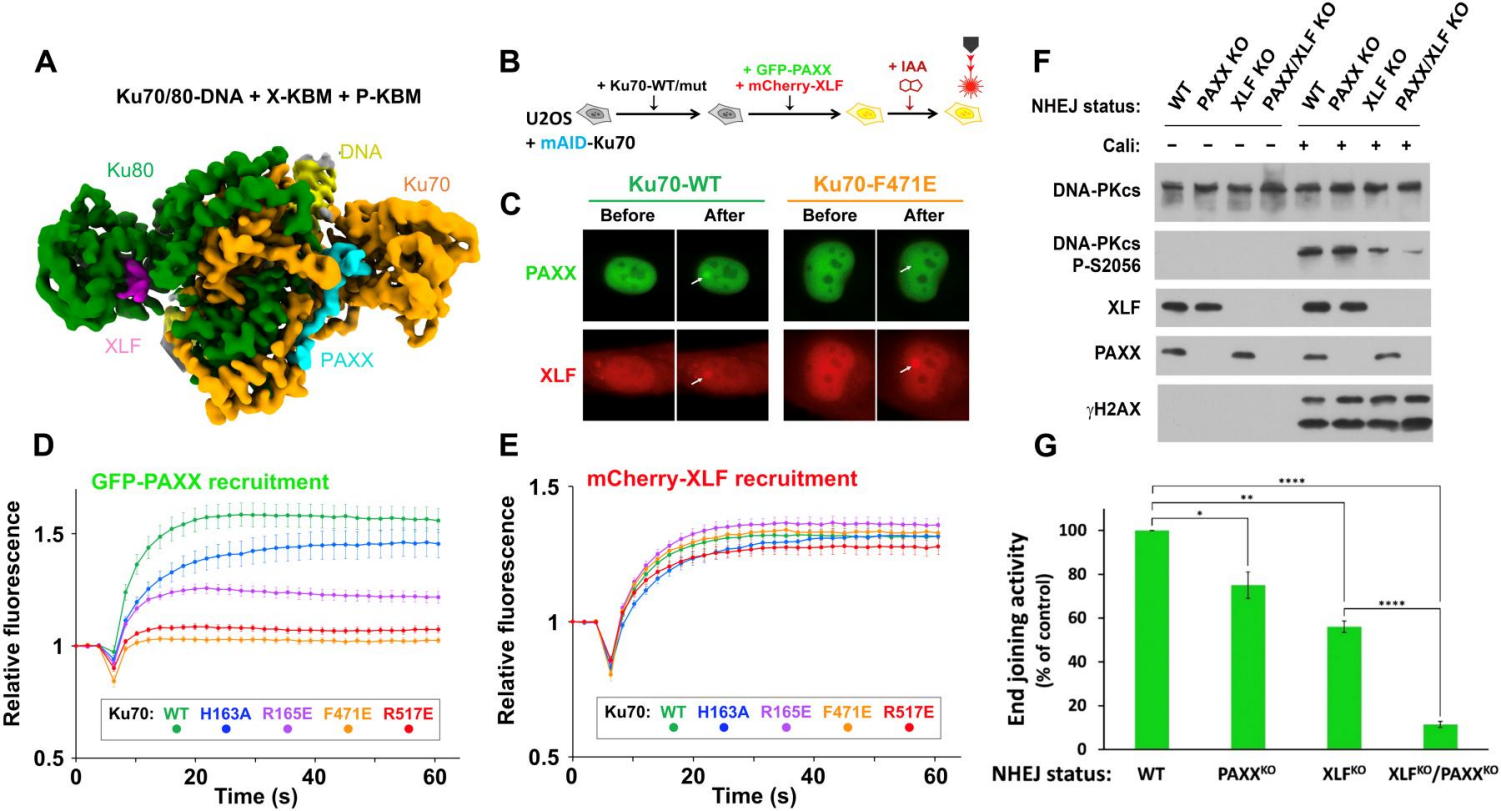
Simultaneous binding of PAXX and XLF to Ku70/80. (**A**) Cryo-EM map (2.68-Å overall resolution) of Ku70/80-DNA with the X-KBM peptide from XLF shown in pink and the P-KBM peptide from PAXX shown in cyan. Ku70 is in orange, Ku80 in green, and DNA in yellow. (**B**) Principle of the laser microirradiation experiment and (**C**) representative images before and after irradiation of nuclei from U2OS cells expressing WT or mutated Ku70, cotransfected with GFP-PAXX and mCherry-XLF. (**D** and **E**) Quantification of fluorescence accumulation at laser-induced DNA damage sites in cells expressing GFP-PAXX and mCherry-XLF. Results were plotted as mean values ± SEM. Ku70 mutants are colored accordingly. (**F**) Western blotting on whole cell extracts from HEK-293T cells as stated, treated or not with 150 pM calicheamicin (Cali) for 1 hour. (**G**) Histogram with mean values ± SEM of end joining efficiency in HEK-293T cells as indicated, quantified by fluorescence expression analyzed by flow cytometry 48 hours after cell cotransfection with Cas9-targeted reporter and control circular plasmids. Values were set at 100% for the WT condition and statistical analysis was performed using the unpaired Student’s t-test. The *P* values for PAXXKO vs WT and XLFKO vs WT were 0.0188 (*) and 0.0011 (**), respectively. The *P* values for XLFKO/PAXXKO vs WT and XLFKO/PAXXKO vs XLFKO were <0.0001 (****).

To obtain higher-resolution structural data for the simultaneous binding of the KBMs of PAXX and XLF to Ku, we collected cryo-EM data of the Ku70/80 heterodimer in complex with peptides corresponding to both KBMs. This map, at 2.68-Å overall resolution, shows binding of both peptides in an arrangement similar to the binding of PAXX alone (Fig. 5A and figs. S16 and S17) and previous x-ray data for the binding of the KBM of XLF (*10*). The length of the two KBMs engaged on either Ku70 or Ku80 is considerably different with a short sequence of eight amino acids observed for the KBM of XLF compared to the extended 28-residue KBM of PAXX. These structures clearly indicate a noncompetitive binding of both KBMs, which is expected given the large distance between the interaction sites (approximately 67 Å).

Last, we challenged this conclusion in cells (Fig. 5, B to E). While the recruitment of GFP-PAXX to DNA damage sites was compromised to various extents by all of the Ku70 mutations tested here, recruitment of mCherry-XLF coexpressed in the same cells was preserved. This supports that XLF can bind Ku independently of PAXX, emphasizing the importance of a distinct function of each protein in NHEJ through parallel emergence of different KBMs targeting separate binding sites on Ku.

### PAXX partially compensates for XLF in DNA end synapsis in cells

Synapsis of DNA ends at DSBs is associated with autophosphorylation of DNA-PKcs (*31*). In particular, the extent of DNA-PKcs S2056 phosphorylation in trans at two-ended DSBs (*32*) correlates with the efficiency of end-bridging during NHEJ in cells (*26*, *33*). We found previously that either XLF or Lig4 was necessary for efficient phosphorylation at S2056 on DNA-PKcs (Fig. 5F and fig. S18) (*22*), in agreement with their requirement for optimal end synapsis in single-molecule studies (*9*, *15*). Here, we similarly estimated the contribution of PAXX to end synapsis in human embryonic kidney (HEK) 293T cells by assessing S2056 phosphorylation after drug treatment under conditions of PAXX KO with or without XLF, while KAP1 P-S824 and γH2AX accounted for the integrity of cell DSBs signaling (Fig. 5F and fig. S18 for extended figures). The sole loss of PAXX barely affected on DNA-PKcs S2056 phosphorylation, unlike XLF loss that reduces S2056 as expected (*33*). However, the combined loss of PAXX and XLF exacerbated the deficit in S2056 phosphorylation resulting from XLF loss alone. PAXX-dependent synapsis function can be recovered upon complementation with WT PAXX but not with PAXX bearing a F201A-mutated P-KBM (fig. S19).

We then assessed the respective contribution of XLF and PAXX on end joining activity in HEK-293T cells after transfection with a Cas9-targeted reporter plasmid. Briefly, following Cas9-induced DSBs, expression of GFP fluorescent protein measured by flow cytometry accounts for direct end joining at the double-break site, while expression of mCherry from a cotransfected plasmid reports transfection efficiency (Fig. 5G). Cells lacking PAXX only were moderately deficient in end joining, consistent with the mild sensitivity to infrared, neocarzinostatin, or zeocin reported for PAXX-deficient cells (*13*, *14*, *18*, *19*, *24*). However, PAXX loss in XLF-deficient cells exacerbated their end joining defect, consistent with PAXX partial compensation for XLF in end-bridging as shown above and in cell survival to treatment with DNA-breaking agents (*14*, *18*, *19*, *24*, *26*).

### Multiple structural branches of NHEJ

**Fig. 6.**
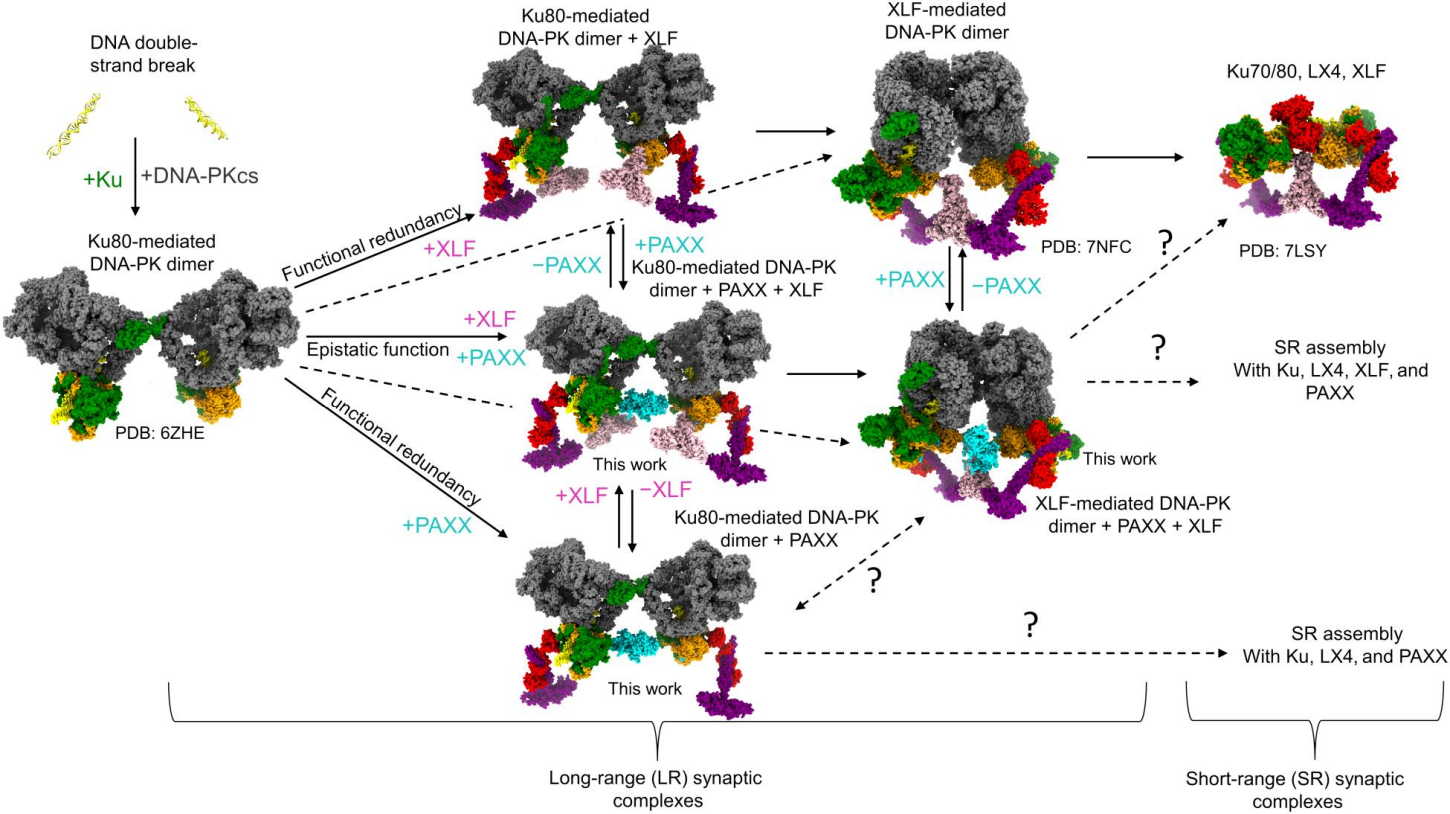
Snapshots of c-NHEJ assembly. A model of the branched pathways and structures of the NHEJ assemblies dependent on the presence of either PAXX or XLF. DNA is in yellow, DNA-PKcs in gray, Ku70 in orange, Ku80 in green, XRCC4 in purple, XLF in pink, Lig4 in red, and PAXX in cyan.

Together, these data allow us to propose a model for the NHEJ mechanism, whereby DNA-PK initially assembles within the Ku80-mediated DNA-PK dimer (Fig. 6). Subsequent complex assembly is then dependent on the recruitment of either XLF or PAXX due to their functionally redundant roles. If only XLF is present (top branch), then it may be able to associate with the Ku80-mediated dimer to allow transition to the XLF-mediated DNA-PK dimer, with or without an intermediate step with one or two Ku-bound XLF molecules. Upon DNA-PKcs removal, XLF-mediated DNA-PK dimer can evolve into the XLF-mediated short-range synaptic complex that has been previously reported (*8*). If only PAXX is available (lower branch), then the Ku80-mediated DNA-PK dimer can be stabilized [Fig. 4 and (*15*)]. It is not yet formally known if this Ku80-mediated DNA-PK/PAXX dimer can transition into a short-range synaptic complex devoid of DNA-PKcs or if a PAXX/DNA-PK dimer resembling an XLF-mediated dimeric form can assemble as an intermediate step. Nevertheless, the fact that, in cells, PAXX can partially compensate for XLF in end joining (Fig. 5) and survival to DNA breaks supports that PAXX is able to stabilize the Ku80-mediated dimer and can somehow substitute for XLF within a short-range synaptic complex. PAXX and XLF have been shown to display epistatic function for the repair of specific DSBs (*20*). If PAXX and XLF are present (middle branch), both Ku80-mediated and XLF-mediated DNA-PK dimers containing PAXX may form (Fig. 5, A to D), evolving possibly into a short-range complex containing XLF as previously described, or a mixed PAXX/XLF complex that has not yet been determined. The simultaneous binding of PAXX and XLF to the same Ku70/80 heterodimer may allow rapid and safe transition from one LR synaptic complex to the other without risk of dismantling the assembly. We show that PAXX defects are not as detrimental for end synapsis and end joining as XLF defects. This could be explained by the interaction of XLF with XRCC4 and additional specific role of XLF in stimulating Lig4 readenylation (*34*). NHEJ appears to have a predominant dependency on the XLF-mediated DNA-PK dimer, whereas PAXX appears to rather stabilize existing DNA-PK dimers and is only partially able to structurally substitute for XLF. During the initial steps of NHEJ, the DNA ends should be maintained at the distance dictated by the LR synaptic assemblies, regardless of the nature of the DSB. This avoids DNA translocation and/or misrepair of DSBs. The structures reported here and the cellular analyses indicate that PAXX through its interaction with Ku70 does not compete with XLF binding to Ku80; rather, it can enhance DNA synapsis during NHEJ.

This work highlights the flexibility of the assembly of the NHEJ machineries and indicates that successful DNA repair may be the result of using a variety of macromolecular assemblies that are more favorable under specific biological conditions or in response to specific types of DNA damage. The role and seeming redundancy of PAXX and XLF in this process have been of debate ever since the discovery of PAXX. Our work helps to resolve this debate, by revealing that each of PAXX and XLF contributes to alternate forms of NHEJ LR synaptic complexes and that this structural redundancy maintains formation of at least one form of LR NHEJ synaptic dimer if either PAXX or XLF function is impaired. Understanding these potentially complex structural pathways used during NHEJ illuminates how the variety of DNA damage events that may be encountered in the cell can be resolved. It is intriguing to consider that these alternate assemblies may be regulated in an inverse manner in specific cancer cells (*27*). Therefore, these findings provide the opportunity to target and modulate specific DNA repair branches while leaving others unihibited, to design more personalized disease-specific therapeutics.

## MATERIALS AND METHODS

### Purification of DNA-PKcs and Ku70/80

DNA-PKcs and full-length His-tagged Ku70/80 were expressed and purified according to (*6*). For crystallography and ITC, the full-length Ku70/80 and a truncated version deleted of its C terminus regions called Ku_ΔC_ [Ku70 (1 to 544)/Ku80 (1 to 555)], were expressed in Sf21 insect cells using a MultiBac expression system. The Ku80 subunit contains a 10xHis-tag, followed by a Tobacco Etch Virus (TEV) protease site on its N terminus. The Ku70/Ku80 heterodimer was purified according to the protocol described in (*10*). The three KuFL variants with single mutation H162A, R165E, or R517E on Ku70 subunits were expressed in insect cells and purified with the same protocol.

### Expression and purification of full-length XLF and LX4

Expression and purification of XLF and Lig4/XRCC4 (LX4) were carried out according to (*7*). Briefly, a construct containing full-length 10xHis-tagged XLF and LX4 were expressed in insect cells. Following expression, cell pellets were resuspended in lysis buffer [20 mM tris (pH 8.0), 5% glycerol, 50 mM KCl, 50 mM NaCl, 5 mM β-mercaptoethanol, 25 mM imidazole, and 2 protein inhibitor cocktail tablets per liter], and cells were sonicated. The resulting lysate was then mixed with 2 μl of benzonase (25 kU of stock, activity per microliter, origin) and MgCl_2_ to a final concentration of 5 mM and left on ice for 20 min. The lysate was then centrifuged (30,000*g* for 20 min at 4°C). The supernatant was purified using Ni–nitrilotriacetic acid resin (QIAGEN) previously equilibrated with lysis buffer and eluted using the lysis buffer containing 300 mM imidazole. Eluted XLF was bound to a Resource Q sepharose anion exchange column in buffer A [20 mM tris (pH 8.0), 50 mM KCl, 50 mM NaCl, 5 mM β-mercaptoethanol, and 1 mM EDTA] and eluted using a linear gradient of buffer A with 850 mM NaCl. Last, the protein was dialyzed into a final buffer of 10 mM tris (pH 8.0), 150 mM NaCl, and 5 mM β-mercaptoethanol before being stored at −80°C for further use (*7*).

### Overexpression and purification of PAXX

Full-length PAXX was expressed and purified according to Ochi *et al.* (*13*). Briefly, PAXX was cloned into a pHAT4 vector and optimized for expression in *Escherichia coli* (*18*). The protein was expressed in BL21(DE3) cells and purified with a 6xHis-tag by Ni-affinity chromatography. The protein was then purified further using cation exchange and size exclusion chromatography.

### DNA annealing

Biotinylated Y-shaped 42- to 55-bp dsDNA were synthesized and annealed as described previously (*35*). The 15-bp double-stranded DNA with a 15-bp 5′ or 3′ overhang were synthesized and annealed as described previously (*14*). Sequences used for annealing are the following: Y-shaped DNA, BiotinCGCGCCCAGCTTTCCCAGCTAATAAACTAAAAACTATTATTATGGCCGCACGCGT (forward) and ACGCGTGCGGCCATAATAATAGTTTTTAGTTTATTGGGCGCG (reverse); 5′ overhang DNA, GATCCCTCTAGATAT (forward) and CGGATCGAGGGCCCGATATCTAGAGGGATC (reverse); 5′ overhang DNA2, GGATCGAGGGCGCGATATCTAGAGGGATC (reverse); 3′ overhang DNA, CGGGCCCTCGATCCG (forward) and CGGATCGAGGGCCCGATATCTAGAGGGATC (reverse).

The oligonucleotides used for crystallization and ITC were resuspended in ultrapure water, and complementary strands were mixed at a 1:1 molar ratio and a final concentration of 500 μM. Then, they were denatured in boiling water for 5 min and cooled down to room temperature overnight. The following oligonucleotides were previously used in (*14*).

The synthetic peptides containing the KBM motif were purchased from Genecust at 95% purity, and the concentrations of the peptide stock solutions were determined by amino acid composition analyses (hydrolysis of peptides and high-performance liquid chromatography analysis). WT and variants of PAXX peptide used for crystallization and ITC are the following: pPAXX_wt, Ac-RRRCPGESLINPGFKSKKPAGGVDFDET-COOH; pPAXX_VFAA, Ac-RRRCPGESLINPGFKSKKPAGGADADET-COOH; pPAXX_N187E, Ac-RRRCPGESLIEPGFKSKKPAGGVDFDET-COOH; pPAXX_S184A, Ac-RRRCPGEALINPGFKSKKPAGGVDFDET-COOH.

### Ku70/80 PAXX cryo-EM complex

Proteins were concentrated using a centricon (Amicon) with a 10-kDa cutoff and buffer exchanged in 20 mM Hepes (pH 7.6), 200 mM NaCl, 0.5 mM EDTA, 2 mM MgCl_2_, and 5 mM dithiothreitol (DTT). Ku was mixed with a 15-bp duplex DNA containing a 5′ 15-bp overhang and PAXX in a 1:1.2:2 ratio. The complex was then incubated with methyl–polyethylene glycol (PEG_4_)–*N*-hydroxysuccinimide (Thermo Fisher Scientific) at a final concentration of 2 mM to reduce the impact of particle orientation bias and incubated for 2 hours at room temperature. The reaction was quenched by adding 0.1 (v/v) of 1 M tris-HCl (pH 8.0).

Aliquots of 3 μl of ~1.7 mg/ml were applied to Holey carbon grids (Quantifoil Cu R1.2/1.3, 300 mesh), glow-discharged for 60 s at a current of 25 mA in PELCO easiGlow (Ted Pella Inc.). The grids were then blotted with filter paper once to remove any excess sample and plunge-frozen, (blotting force of −5 and blotting time of 3 s) in liquid ethane using a FEI Vitrobot Mark IV (Thermo Fisher Scientific) at 4°C and 95% humidity.

### Ku70/80-DNA-PAXX crystallization

For crystallization experiments, the 10xHis-tag of the Ku_ΔC_ heterodimer was cleaved by the TEV protease (Ku_ΔC-noTag_). The 3′ overhang DNA (DNA F) allowed to obtain diffracting crystals. The PAXX KBM peptide is 28 nucleotide oligomers (amino acids 177 to 204). The Ku_ΔC-noTag_-DNA-peptide complexes were performed at a 1:1.2:2 molar ratio respectively with a final concentration of 10 mg/ml for Ku heterodimer. Crystallization screens were performed at the HTX platform (EMBL, Grenoble, PID 12324 iNEXT-Discovery) following the sitting drop method and with visualization at 4°C. The crystals were reproduced and optimized in the laboratory at 17°C using the hanging drop method and by mixing 1 μl of the protein solution (10 mg/ml) with 1 μl of the crystallization solution [12% PEG 3350, 0.1 M bis-tris propane (pH 8.5), and 0.3 M ammonium sulfate]. Single crystals (200 μm by 400 μm by 75 μm) grew in 7 to 10 days and were frozen in 10% glycerol.

### Cryo-EM of DNA-PK, LX4, and PAXX complex

Proteins were concentrated using a centricon (Amicon) with a 30-kDa cutoff and buffer exchanged into 20 mM Hepes (pH 7.6), 200 mM NaCl, 0.5 mM EDTA, 2 mM MgCl_2_, and 5 mM DTT. Purified Ku70/80 full length was then first mixed with Y-shaped 42- to 55-bp DNA before being mixed with purified DNA-PKcs, LX4, and PAXX in a 2:2:2:2 ratio respectively.

Aliquots of 3 μl of the NHEJ super complex (~2.5 mg/ml) were mixed with 8 mM CHAPSO to eliminate particle orientation bias (final concentration; Sigma-Aldrich) before being applied to Holey carbon grids (Quantifoil Cu R1.2/1.3, 300 mesh), glow-discharged for 60 s at a current of 25 mA in PELCO easiGlow (Ted Pella Inc.). The grids were then blotted with filter paper once to remove any excess sample and plunge-frozen in liquid ethane using a FEI Vitrobot Mark IV (Thermo Fisher Scientific) at 4°C and 95% humidity.

### Cryo-EM data acquisition

Both datasets were collected on a Titan Krios with the PAXX/Ku complex collected at the Department of Materials Sciences, University of Cambridge, and the PAXX–DNA-PK supercomplex in the Department of Biochemistry, University of Cambridge. All data collection parameters are given in table S1.

### Cryo-EM image processing

The classification process for the two datasets is summarized schematically in fig. S1. The final reconstructions obtained had overall resolutions (table S1), which were calculated by Fourier shell correlation at 0.143 cutoff.

### Cryo-EM structure refinement and model building

The model of Ku70/80 (PDB: 1JEY) was used as an initial template and rigid body fitted into the cryo-EM density. The DNA was manually built on the basis of 1JEY with the 5′ overhang sequence (see above). The C-terminal PAXX peptide was manually built on the basis of the sequence, the density-modified map, and the peptide present within the super complex monomer and dimer.

The model of the DNA-PK monomer and dimer (PDB: 7NFE and 7NFC) were used as initial templates and rigid body fitted into the cryo-EM density for the super complex monomer and dimer with PAXX in UCSF chimera (*36*) and manually adjusted and rebuilt in Coot (*37*). Extra density for LX4 and PAXX was docked using PDB 3II6 and PDB 3WTD in UCSF chimera (*36*) and, again, manually adjusted and rebuilt in Coot (*37*). The PAXX peptide was fitted according to the Ku70/80 PAXX alone structure [PDB: 7ZVT (this study)]. The PAXX peptide linker and globular head domain were docked into the super complex DNA-PK dimer structure but were not subject to final refinement due to the unclear density.

### PAXX fitting

The EM map of the NHEJ super complex was used to manually fit the PAXX homodimer (PDB: 3WTD) at the interface between Ku dimer. The fit was further refined using ChimeraX “Fit in Map” tool. The EM densities around 5 Å of the atoms of the PAXX fit was extracted using the “volume zone” command in ChimeraX (*38*). ADM_EM program (*30*) was used to perform a full exhaustive rigid body search to identify the optimum fit of the PAXX homodimer. The program generated 431 optimum fits out of 1000 requested solutions. The cross-correlation coefficient (CCC) scores between the fits and the zoned density map were normally distributed with mean and SD of 0.243 and 0.013, respectively (fig. S12A). Note that the rigid body fitting program does not consider the twofold symmetry found in the NHEJ super complex. Because of this, many of the best scoring fits of PAXX will not be meaningful within the context of the symmetry of the super complex. Of 431 best fits, the PAXX fit with rank 37 was selected as the most likely fit (fig. S14B). This was justified on the basis of the manual observation that the twofold axis of the PAXX homodimer approximately aligned with the twofold axis of the DNA-PKcs homodimer (fig. S12C). The CCC score of 0.262 for the selected fit was substantial compared to the mean score of the distribution. The *z* score for the selected fit was 1.38.

### Crystallography data acquisition—Processing and refinement

Diffraction data were collected at the Proxima1 beamline at synchrotron SOLEIL (St. Aubin). The dataset was indexed and integrated using the XDS package (*39*), the XDSME package (XDS Made Easier, https://github.com/legrandp/xdsme) and the CCP4 suite (*40*). The x-ray data present an anisotropic diffraction (between 2.82 and 6.14 Å). The anisotropy was corrected using the STARANISO server (Staraniso GlobalPhasing, https://staraniso.globalphasing.org) or autoPROC (*41*). After anisotropy correction, the dataset is at a 2.97-Å resolution for Ku-(DNA F)-PAXX. The structure was resolved by molecular replacement, with the Phaser Phenix software (https://phenix-online.org), using the previously resolved structure of the Ku heterodimer bound to a hairpin DNA (PDB: 1JEY) (*42*). The DNA molecule and Ku70 vWA domain were omitted and built using the Coot program (CCP4 package). Four Ku molecules were placed in the asymmetric unit. After refinement using Phenix or Buster [global Phasing, Bricogne G., Blanc E., Brandl M., Flensburg C., Keller P., Paciorek W., Roversi P, Sharff A., Smart O.S., Vonrhein C., Womack T.O. (2017). BUSTER version 2.10.4. Cambridge, United Kingdom: Global Phasing Ltd.], an electron density was observed on the Ku70 vWA allowing to position it. The PAXX peptide was built into the density using Coot.

### Isothermal calorimetry measurements

Interaction between Ku-DNA complex and the different peptides (WT and variants) was measured by isothermal calorimetry using a VP-ITC machine. For ITC measurements, the full-length Ku heterodimer was used with the DNA G and F as previously used in (*14*). Before measurements, the Ku heterodimer was extensively dialyzed against buffer [20 mM tris (pH 8), 150 mM NaCl, and 5 mM β-mercaptoethanol]. The cell reaction (1.8 ml) was loaded with the preformed Ku-DNA complex at a 1:1.2 molar ratio respectively. Ku was loaded at 5 μM concentration. The syringe (300 μl) was loaded with the different peptides at different concentrations ranging between 50 and 100 μM. The Ku-DNA complex was titrated by automatic injections of 10 μl at 25°C. Stoichiometry of the reaction (*N*) and association constant (*K*; M^−1^) were generated by nonlinear least-squares fitting of the experimental data using the single set of independent binding sites model of the Origin software provided with the instrument (OriginLab, MicroCal analysis). The same experiments were performed with the three variants of Ku.

### Nanodifferential scanning fluorimetry

Nanodifferential scanning fluorimetry measurements were performed on a Tycho NT.6 device (NanoTemper) with Ku_FL_ dialyzed against buffer 20 mM tris HCl (pH 8), 150 mM NaCl, and 5 mM β-mercaptoethanol. We used 9 μl of Ku_FL_ or mutants at 5 μM in absence or presence of DNA at 7.5 μM to obtain a (1:1.5) molar ratio. Each point corresponds to an average of three measurements. The temperature of transition that corresponds to the change of environment of the tryptophan is deduced from the first inflexion point of the 330:350 nm ratio versus temperature.

### Cell lines, cell culture, and cell engineering

U2OS human osteosarcoma cells (European Collection of Authenticated Cell Cultures, Salisbury, United Kingdom) and HEK-293T human embryonic cells were grown in Dulbecco’s modified Eagle’s medium (Eurobio, France) supplemented with 10% fetal bovine serum (Eurobio, France), penicillin (125 U/ml), and streptomycin (125 μg/ml). Cells were maintained at 37°C in a 5% CO_2_-humidified incubator.

U2OS cells knocked out for PAXX were obtained following cell transfection with the pCAG-eCas9-GFP-U6-gRNA-PAXX vector (see below) using Lipofectamine 2000 (Thermo Fisher Scientific) as a transfection reagent. Following cell sorting, individual clones were isolated and checked by Western blot. HEK-293T cells knocked out for PAXX, XLF, DNA-PKcs, and Lig4 were generated in a similar manner by transfection with the corresponding pCAG-eCas9-GFP-U6-gRNA vectors using jetPEI (Polyplus) as a transfection reagent.

The generation of U2OS cells expressing a mAID-Ku70 in place of the endogenous protein was achieved as follows. First, U2OS cells were cotransduced with two lentiviral constructs allowing both the expression of an shRNA against Ku70 and the expression of a Ku70 protein resistant to the shRNA and fused to a mAID tag at its N terminus. The latter vector also expresses a puromycin resistance gene, enabling the selection of a transduced cell population. Second, these cells were further transduced with a construct expressing the rice Transport Inhibitor Response 1 (TIR1) protein, and individual clones were isolated and functionally screened for the loss of viability upon treatment with auxin (IAA). Positive clones were checked by Western blot to confirm the loss of endogenous Ku and the degradation of mAID-Ku70 in response to IAA.

Production of lentiviral particles in HEK-293T cells and transduction of U2OS cells were performed as previously described (*43*).

### Multiphoton laser microirradiation

Live cell microscopy and laser microirradiation were conducted as previously described (*43*).

### End joining assay

Briefly, HEK-293T cells were seeded in six-well plates and transfected 24 hours later with a mix of Cas9-targeted reporter substrate, Cas9/gRNA-expressing vector, and pmCherry control plasmid. Cells were trypsinized 2 days after transfection, washed with phosphate-buffered saline (PBS), and analyzed by flow cytometry on a LSRFortessa X-20 cell analyzer (BD Biosciences). The integrated green fluorescence signal accounting for end joining repair events (% positive cells × mean fluorescence) was normalized to that of transfection control (mCherry).

### Plasmids and DNA manipulations

A FLAG-tagged shRNA-resistant Ku70 lentiviral expression vector was derived from the previously described pLV3-FLAG-Ku70 vector (*10*) by overlap extension polymerase chain reaction (PCR) mutagenesis using Kpn2-FLAG-F and Mlu-Ku70-R as outer primers and Ku70-shR-F and Ku70-shR-R as inner primers that introduce 10 silent point mutations in the shRNA target sequence of Ku70 (codons Q52-T58). The resulting fragment was subcloned into the Kpn 2I and Mlu I restriction sites of the pLV3 vector backbone. The GFP-tagged version of FLAG-Ku70-shR-WT expressing vectors were obtained by PCR amplification of the enhanced GFP (EGFP) coding sequence from the pEGFP-C1 plasmid (Clontech) using the Pme-GFP-F and pme-GFP-R primers. The PCR fragment was then inserted at the Pme I restriction site of pLV3-FLAG-Ku70-shR-WT vector using the Hot-Fusion strategy (*44*) to generate the pLV3-EGFP-FLAG-Ku70-shR-WT lentiviral vector.

The various expression vectors for mutant forms of Ku70 were obtained in a similar manner following an additional step of overlap extension PCR mutagenesis with the corresponding Ku70-mut-F and Ku70-mut-R oligonucleotides as mutated inner primers (see below the list of primers).

The different GFP-tagged PAXX constructs were obtained by PCR amplification of the PAXX cDNA with the external primers Kpn2-PAXX-F and pLV-R and preceded for PAXX mutants by an additional overlap extension PCR mutagenesis step with the corresponding PAXX-mut-F and PAXX-mut-R oligonucleotides as the internal mutated primers (see below the list of primers). All PAXX PCR fragments were inserted into the Kpn 2I and Bcu I restriction sites of the pLV3-EGFP-FLAG-Ku70-WT plasmid in place of the FLAG-Ku70 cDNA.

The pLV3-mCherry-XLF vector was obtained by replacing the cyan fluorescent protein (CFP) coding sequence of pLV3-CFP-XLF (*10*) by the mCherry coding sequence following. To this end, the pLV3-CFP-XLF vector was digested with Pme I and Kpn 2I and a mCherry cDNA fragment resulting from PCR amplification from the pmCherry-NLS plasmid (a gift from Martin Offterdinger; Addgene plasmid #39319; http://n2t.net/addgene:39319; RRID: Addgene_39319) with primers mCh-pme-F and Kpn2-mCh-R was inserted using the Hot-Fusion strategy (*44*).

To knockdown Ku70 expression, the pLVTHM2-shKu70 lentiviral vector was generated by inserting between the Mlu I and Cla I restriction sites of pLVTHM2 (*10*) the preannealed oligonucleotides shKu70-F and shKu70-R, allowing the expression of an shRNA against codons Q52-T58 of Ku70 (*43*, *45*).

A lentiviral vector expressing a PuroR-T2A-mAID-Ku70-shR construct was generated by inserting first a T2A cassette (preannealed oligonucleotides kpn2-T2A-Mlu-F and kpn2-T2A-Mlu-R) into the Kpn 2I and Mlu I restriction sites of the pLV3 plasmid (*10*). A PCR-amplified fragment encoding a Puro-resistant cDNA (PCR reaction with primers HF-Puro-F and HF-Puro-R on a synthetic DNA molecule as a template) was then added by Hot-Fusion (*44*) at the Kpn 2I site. The cDNA of the mAID (*28*) was inserted between Xma I and Eco RI restriction sites of the previous plasmid following PCR amplification using primers Xma-mAID-F and Eco-mAID-R and the pAID1.1-N plasmid (BioROIS, Japan) as a template. Last, the resulting vector was digested with Bam HI/Mlu I to insert a fragment encoding Ku70-shR excised from pLV3-FLAG-Ku70-shR-WT to give the pLV3-PuroR-T2A-mAID-Ku70-shR plasmid.

The rice TIR1 cDNA fused to a sequence encoding three copies of c-Myc tag was PCR-amplified from the pAID1.1-N plasmid (BioROIS, Japan) with Kpn2-TIR1-F and Mlu-Myc-R primers. The resulting PCR fragment was inserted into the Kpn 2I and Mlu I restriction sites of the pLV3 lentiviral vector (*10*).

PAXX KO was achieved using the CRISPR-Cas9 technology by inserting the preannealed PAXX-gRNA-F and PAXX-gRNA-R oligonucleotides into the Bbs I restriction sites of pCAG-eCas9-GFP-U6-gRNA plasmid (a gift from J. Zou, Addgene plasmid #79145; http://n2t.net/addgene:79145; RRID: Addgene_79145) to obtained the pCAG-eCas9-GFP-U6-gRNA-PAXX vector. XLF, DNA-PKcs, and Lig4 KOs were performed in the same manner with the following pairs of oligonucleotides: XLF-gRNA-F/XLF-gRNA-R, PKcs-gRNA-F/PKcs-gRNA-R, and Lig4-gRNA-F/Lig4-gRNA-R, respectively.

### Drug treatment, protein extraction, separation, and detection

Stock solution of calicheamicin γ1 (Cali), gift from P. R. Hamann (Wyeth Research, Pearl River, NY, USA), was made at 40 μM in ethanol and stored at −20°C. For drug exposure, exponentially growing cells in 60-cm-diameter dishes were either mock-treated or treated with Cali in fresh medium for 1 hour at 37°C. Then, cells were washed with PBS and trypsinized. Pellets were fractionated as follows. Cells were first resuspended for 7 min on ice in 120 μl of extraction buffer 1 [50 mM Hepes (pH 7.5), 150 mM NaCl, and 1 mM EDTA] containing 0.1% Triton X-100 and supplemented with the Halt protease and phosphatase inhibitor cocktail (Thermo Fisher Scientific) with intermittent gentle vortexing. Following centrifugation at 14,000 rpm for 3 min, the supernatant was removed, and pellets were gently resuspended with pipette tips in 120 μl of extraction buffer 2 [50 mM Hepes (pH 7.5), 75 mM NaCl, and 1 mM EDTA] containing 0.025% Triton X-100 and 30 U of ribonuclease A/T1 (Thermo Fisher Scientific), incubated for 15 min at 25°C under agitation, and centrifuged as above. Pellets were resuspended in 120 μl of lysis buffer [50 mM tris (pH 8.1) and 10 mM EDTA] supplemented with 0.1% Triton X-100 and 0.3% SDS and sonicated (Vibracel, Bioblock Scientific) or passed 10 times through a 26-gauge needle. For whole cell extracts preparation, PBS-washed cell pellets were directly resuspended in lysis buffer as above and passed 10 times through a 26-gauge needle. Protein content was measured with BCA reagent (Pierce), and equivalent protein amounts were denaturated in loading buffer at 1× final concentration [50 mM tris-HCl (pH 6.8), 10% glycerol, 1% SDS, 300 mM 2-mercaptoethanol, and 0.01% bromophenol blue] at 95°C for 5 min, separated on SDS–polyacrylamide gel electrophoresis gels (Bio-Rad; 4 to 15% Tris-Glycine eXtended) precast gels) before overnight transfer onto Immobilon-P polyvinylidene difluoride (Millipore) membranes. Staining with AdvanStain Iris (Advansta) controlled homogeneous loading and prestained protein ladder allowed cutting the membrane for separate and simultaneous blotting with selected antibodies. Membranes pieces were blocked for 60 min with 5% nonfat dry milk in PBS and 0.1% Tween 20 (Sigma-Aldrich) (PBS-T buffer), incubated as necessary with primary antibody diluted in PBS-T containing 1% bovine serum albumin (immunoglobulin- and lipid-free fraction V; Sigma-Aldrich), and washed three times with PBS-T; membranes were incubated for at most 1 hour with horseradish peroxidase–conjugated secondary antibodies in PBS-T and washed three times with PBS-T. Immunoblots were visualized using autoradiography films together with enhanced chemiluminescence (WesternBright ECL, Advansta).

### Antibodies

For immunoblotting, the following were used: mouse monoclonal antibodies: anti–β-actin (clone AC-15, Ambion), anti-Ku80 (clone 111), Ku70 (clone N3H10), and DNA-PKcs (clone 18.2) (Thermo Fisher Scientific); rabbit polyclonal antibodies: anti-KAP-1 PhS824 (IHC-00073, Bethyl Laboratories), anti-XLF (A300-730A, Bethyl Laboratories, or A199957, Abclonal), anti-LIG4 (A11432, Abclonal), anti-DNA-PKcs PhS2056 (ab18192), KAP-1 (ab10483), anti-PAXX (ab126353) (Abcam), and anti-γH2AX (Cell Signaling Technologies); peroxidase-conjugated goat anti-mouse or anti-rabbit secondary antibodies were from Jackson ImmunoResearch Laboratories.

Oligonucleotides (DNA linkers and PCR primers) for in vivo analysis are shown as follows:

**Eco-mAID-R** ctctcGAATTCCACGCGTcctaggtGGATCCGCTTTTATACATCCTCAAATCGATTTTCCTC

**HF-Puro-F** GCCTCGAGGTTTAAACTACGGgatcTCCGCcATGACaGAGTACAAGCCaACaGTG

**HF-Puro-R** CCGCATGTTAGCAGACTTCCTCTGCCCTCGGCACCtGGCTTtCtGGTCATGCACC

**Kpn2-FLAG-F** ctctcgTCCGGAgccgccaccATGGACTACAAGGATG

**Kpn2-mCh-R** GTTTTTCATTTGAGCTCGAGATCTGAGTCCGGActtgtacagctcgtcc

**Kpn2-PAXX-F** CTGTACAAGTCCGGACTCAgatccatggacccgc

**Kpn2-Tir1-F** ctctcTCCGGAgccgccaccATGACGTACTTCCCGGAGGAGGTG

**kpn2-T2A-Mlu-F** CCGGAGGGCAGAGGAAGTCTGCTAACATGCGGTGACGTCGAGGAGAATCCTGGACCCGGGtcactcA

**kpn2-T2A-Mlu-R** CGCGTgagtgaCCCGGGTCCAGGATTCTCCTCGACGTCACCGCATGTTAGCAGACTTCCTCTGCCCT

**Ku70-F471-F** CGTTGAGAAGCTTCGCgagACATACAGAAGTGACAGCTTTGAGAACCCC

**Ku70-F471-R** CTGTCACTTCTGTATGTctcGCGAAGCTTCTCAACGATAGCCTTCATCTTG

**Ku70-H163A-F** CAATTCAAGATGAGTgcTAAGAGGATCATGCTGTTCACCAATGAAGAC

**Ku70-H163A-R** GCATGATCCTCTTAgcACTCATCTTGAATTGGACATCACTAAAGAGG

**Ku70-R165E-F** CAAGATGAGTCATAAGgaGATCATGCTGTTCACCAATGAAGACAACCC

**Ku70-R165E-R** GAACAGCATGATCtcCTTATGACTCATCTTGAATTGGACATCACTAAAG

**Ku70-R517E-F** GTTGAAGCAATGAATAAAgaACTGGGCTCCTTGGTGGATGAGTTTAAGG

**Ku70-R517E-R** CCACCAAGGAGCCCAGTtcTTTATTCATTGCTTCAACCTTGGGCAATGTC

**Ku70-shR-F** CTCAatccGAgGAcGAacTcACcCCTTTTGACATGAGCATCCAGTGTATCC

**Ku70-shR-R** GGgGTgAgtTCgTCcTCggatTGAGATTCAAACATAGCCTTGGAGG

**Lig4-gRNA-F** caccGTTCAGCACTTGAGCAAAAG

**Lig4-gRNA-R** aaacCTTTTGCTCAAGTGCTGAAC

**mCh-pme-F** CGATCACGAGACTAGCCTCGAGGTTTgccaccatggtgagcaagggcg

**Mlu-Ku70-R** ctctgcACGCGTCAGTCCTGGAAGTGCTTGGTGAGGGC

**Mlu-Myc-R** ctctcACGCGTctATCCGTTCAAGTCTTCTTCTGAGATTAATTTTTG

**PAXX-F201A-R** cctctcACTAGTtaggtctcatcgGCgtccacgccaccagctggtttcttac

**PAXX-gRNA-F** caccgTGACCGACGCCGCGGAGCTT

**PAXX-gRNA-R** aaacAAGCTCCGCGGCGTCGGTCAc

**PAXX-N187E-F** GTCCTGGTGAAAGCTTGATAgAgCCCGGGTTCAAGAGTAAGAAACC

**PAXX-N187E-R** GGTTTCTTACTCTTGAACCCGGGcTcTATCAAGCTTTCACCAGGAC

**PAXX-S184A-F** CAGGAGGCGGTGTCCTGGTGAAgcCTTGATAAACCCCGGGTTCAAG

**PAXX-S184A-R** CTTGAACCCGGGGTTTATCAAGgcTTCACCAGGACACCGCCTCCTG

**PAXX-V199A-R** cctctcACTAGTtaggtctcatcgaagtccGcgccaccagctggtttcttac

**PKcs-gRNA-F** caccGGTACCCACCCAGCACCGCG

**PKcs-gRNA-R** aaacCGCGGTGCTGGGTGGGTACC

**Pme-GFP-F** CGATCACGAGACTAGCCTCGAGGTTTAAACGCCACCATGGTGAGCAAGGGC

**pme-GFP-R** ggtgcggcTCCGGAgatcCCGTAgtttGGACTTGTACAGCTCGTCCATGCCG

**pLV-R** CCAGTCAATCTTTCACAAATTTTGTAATCCAGAGG

**shKu70-F** CGCGTCCCCGAGTGAAGATGAGTTGACATTCAAGAGATGTCAACTCATCTTCACTCTTTTTGGAAAT

**shKu70-R** CGATTTCCAAAAAGAGTGAAGATGAGTTGACATCTCTTGAATGTCAACTCATCTTCACTCGGGGA

**XLF-gRNA-F** caccGGAGATTATCCAAATGACAG

**XLF-gRNA-R** aaacCTGTCATTTGGATAATCTCC

**Xma-mAID-F** ctcactCCCGGGTCCAAGGAGAAGAGTGCTTGTCCTAAAG

## Supplementary Material

20230531-1

## Supplementary Materials

### This PDF file includes:

Figs. S1 to S19 Tables S1 to S2 Legend for movie S1 References
