# Supplementary material for: PAXX binding to the NHEJ machinery explains functional redundancy with XLF
Source: Sci Adv. 2023 May 31;9(22):eadg2834. doi: 10.1126/sciadv.adg2834 (PMC10413649; doi:10.1126/sciadv.adg2834)
Supplement: Supplementary file 2 — Figs. S1 to S19 Tables S1 to S2 Legend for movie S1 References [file sciadv.adg2834_sm.pdf]

Science Advances
AAAS

Supplementary Materials for

PAXX binding to the NHEJ machinery explains functional redundancy with XLF

Murielle Seif-El-Dahan *et al.*

Corresponding author: Amanda K. Chaplin, ac853@leicester.ac.uk;
Jean-Baptiste Charbonnier, jb.charbonnier@cea.fr; Patrick Calsou, calsou@ipbs.fr;
Virginie Ropars, virginie.ropars@cea.fr

Sci. Adv. **9**, eadg2834 (2023)
DOI: 10.1126/sciadv.adg2834

The PDF file includes:

Figs. S1 to S19
Tables S1 and S2
Legend for movie S1
References

Other Supplementary Material for this manuscript includes the following:

Movie S1

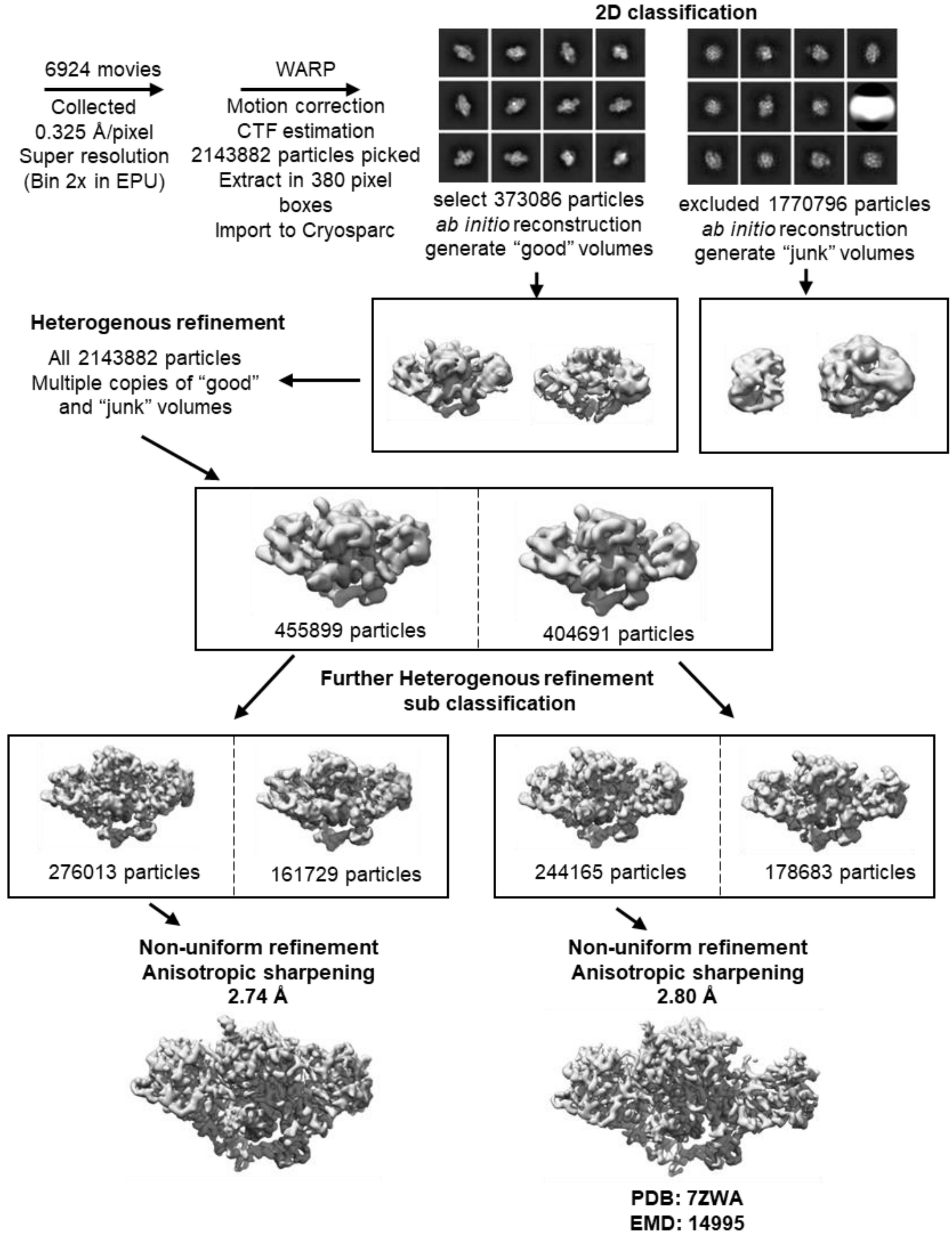

Fig. S1.

Single-particle cryo-EM image processing workflow for Ku70/80-DNA with and without PAXX. Schematic showing particle picking using WARP and processing including 2D classification and *ab initio* reconstruction using CryoSPARC. The two main classes generated with the corresponding number of particles is shown and the final two maps following non-uniform refinement with resolutions for an FSC of 0.143 are given.

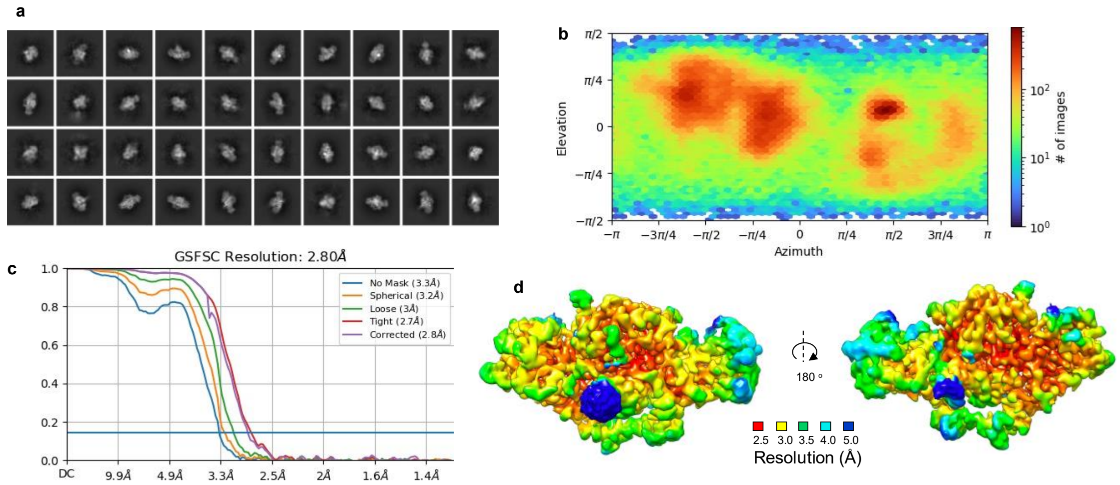

Fig. S2.

Cryo-EM data of Ku70/80-DNA with PAXX. a) Examples of 2D classes. **b)** Angular distribution calculated in cryoSPARC for particle projections shown as a heat map. **c)** FSC resolution curves and viewing distribution plot. **d)** Local resolution map of the Ku-DNA with PAXX cryo-EM map. The colours corresponding to each resolution are displayed on the specific key chart below the maps.

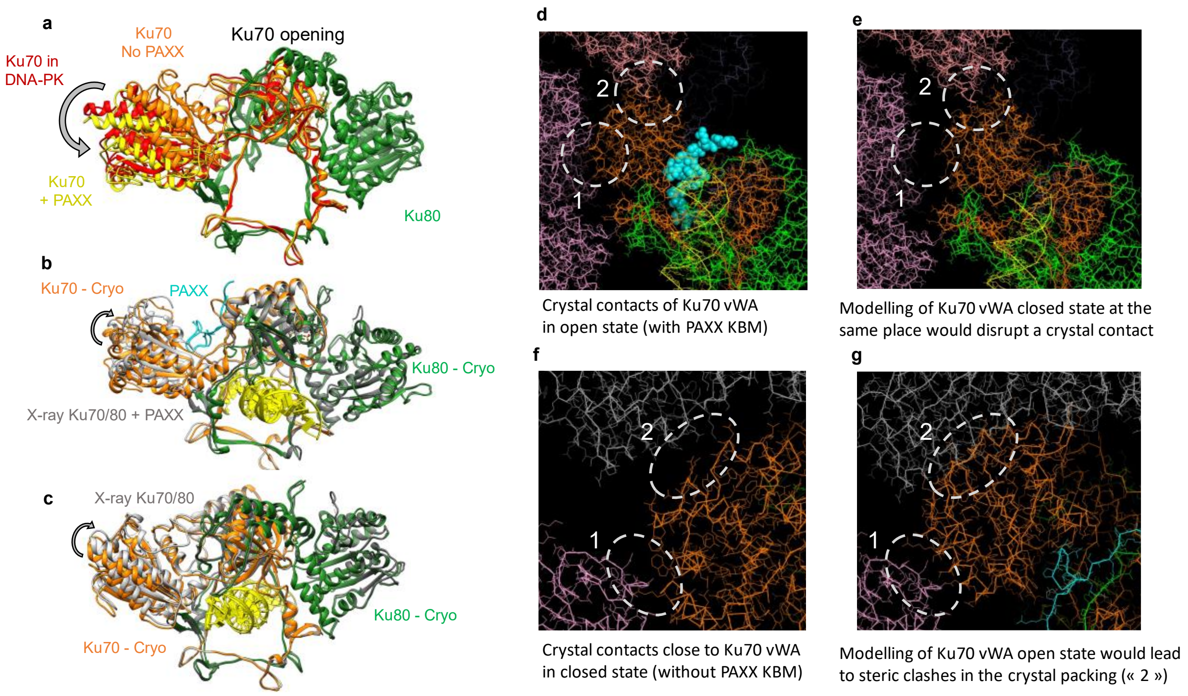

Fig. S3.

Open and closed vWA of Ku70 upon PAXX binding and the X-ray crystallographic contacts. **a)** An overlay of Ku70/80 with no PAXX bound with Ku70 in orange, Ku70/80 in DNA-PK with Ku70 in red and Ku70 with the P-KBM of PAXX bound with Ku70 in yellow. Ku80 is always shown in green and DNA removed for clarity to show the clear openings of Ku70. **b)** An overlay of the cryo-EM structure of Ku70/80 (orange and green) with PAXX bound (cyan) with the X-ray crystallography structure with PAXX bound (shown in grey) with the DNA shown in yellow. **c)** An overlay of the cryo-EM structure of Ku70/80 with no PAXX bound (orange and green) with the X-ray crystallography structure with no PAXX bound (grey) with the DNA in yellow. **d)** The vWA domain of Ku70 in the open state (bound to PAXX KBM (cyan)) makes crystal contacts with Ku70/80 symmetric molecules in the crystal (in pink (1), in salmon (2), and in light blue in background). **e)** Positioning the closed state of Ku70 vWA at this position indicates that it would disrupt a major crystal contact (noted « 1 »). **f)** The vWA domain of Ku70 in the closed state makes crystal contacts with Ku symmetric molecules in the crystal (in pink (1) and in grey (2)) **g)** Positioning the open state of Ku70 vWA at this position indicates that it would make steric clash in the crystal packing (noted « 2 »). This observation and the similarity of the cryo-EM and crystal structures suggests that the open and closed conformation are present in solution. During crystal growth, the two forms are selected and make specific crystal contacts.

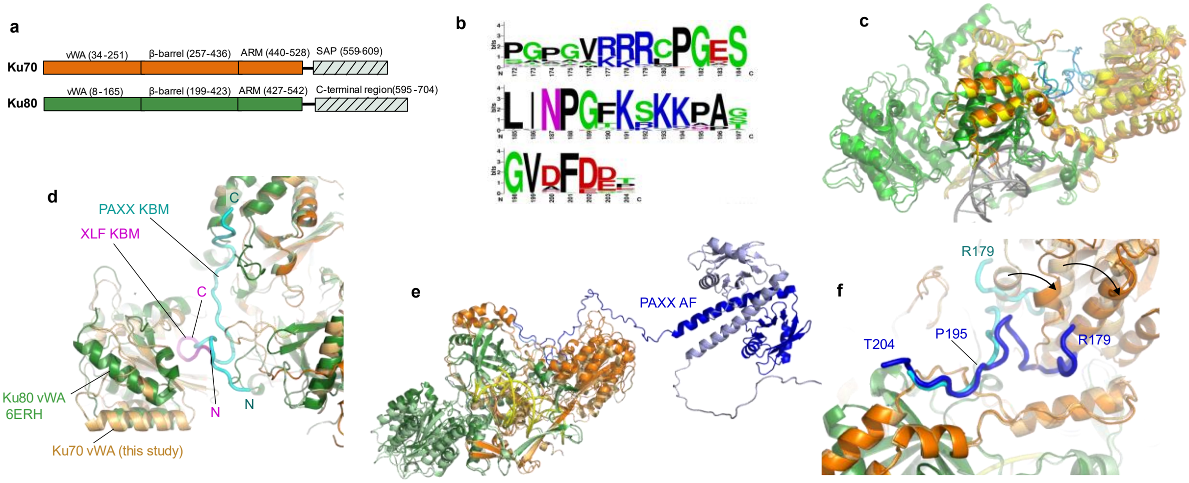

Fig. S4.

a) Scheme of Ku70 and Ku80 domains. $Ku_{\Delta C}$ is a construct deleted of both Ku70 and Ku80 C-termini (Ku70 1-544, Ku80 1-551). **b)** Logo motif of the PAXX KBM, motif obtained from multiple sequence alignments of these proteins as indicated (*46*). **c)** Superposition of the cryo-EM (dark colours) and crystal structures (light colours) of the Ku70/80/DNA complex bound to the KBM of PAXX (rmsd of 1,05Å over 910 Cα). **d)** Superposition of Ku80 (dark green) with XLF KBM (magenta) with Ku70 (light orange) bound to PAXX KBM (cyan) (rmsd of 2,6Å over 151 Cα). The N-terminus and C-terminus of the XLF and PAXX KBM are indicated. **e)** Alphafold-Multimer (AF) model of Ku70/80/PAXX protein interactions. Ku70/80 model of AF (light colours) is superimposed with the cryo-EM of Ku-DNA-PAXX(KBM) (dark colour) (rmsd of 0,58Å over 231 Cα). The PAXX dimer modelled by AF is shown in light and dark blue. **f)** AF's model of PAXX KBM superimposes well with the PAXX KBM observed in the cryo-EM structure on the C-terminal part (P195 to T204). The N-terminal part prediction (R179-P195) since AF does not predict an opening of the Ku70 vWA region (arrows).

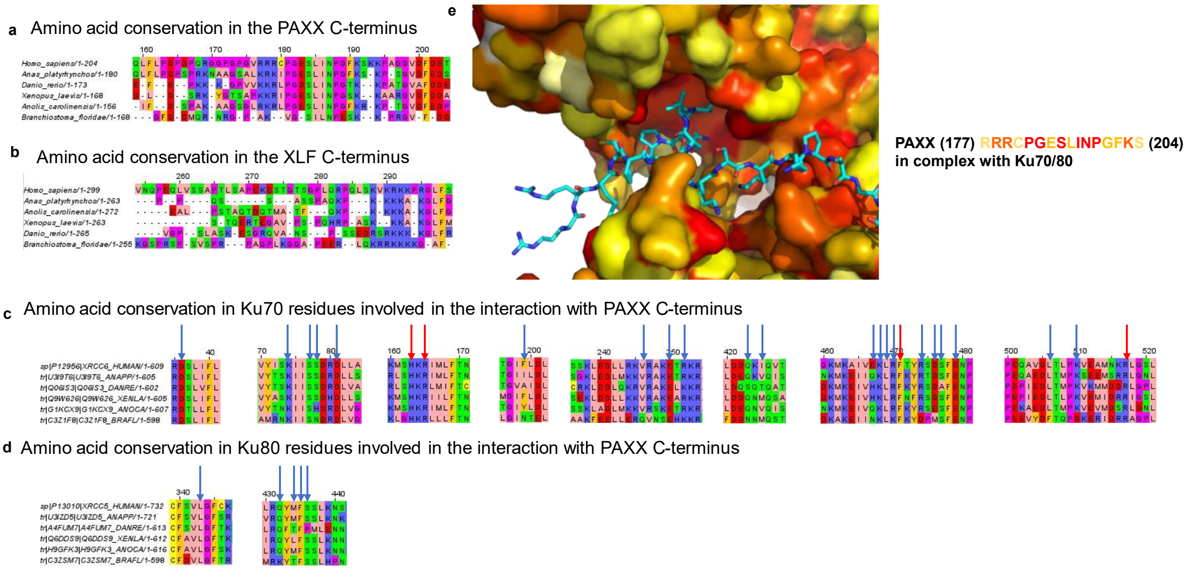

Fig. S5.

a) Zoom of the alignment of sequences of metazoan PAXX on the P-KBM regions. Coloured by type of amino acids **b)** Zoom of alignment of metazoan XLF in the X-KBM region c-d) Zooms of the alignments of metazoan sequences of **c)** Ku70 and **d)** Ku80 in the regions of Ku70 and Ku80 involved in the interaction with PAXX KBM. Blue arrows are positions in contact at 4 Å in X-ray structure. Red arrows are positions in contact at 4 Å in X-ray structure and mutated **e)** Surface representation of conserved residues of Ku in contact with P-KBM generated with Consurf. (right) Sequence of the C-terminus of PAXX coloured according to the conservation of the amino acids (from red (highly conserved) to pale yellow and white (not conserved).

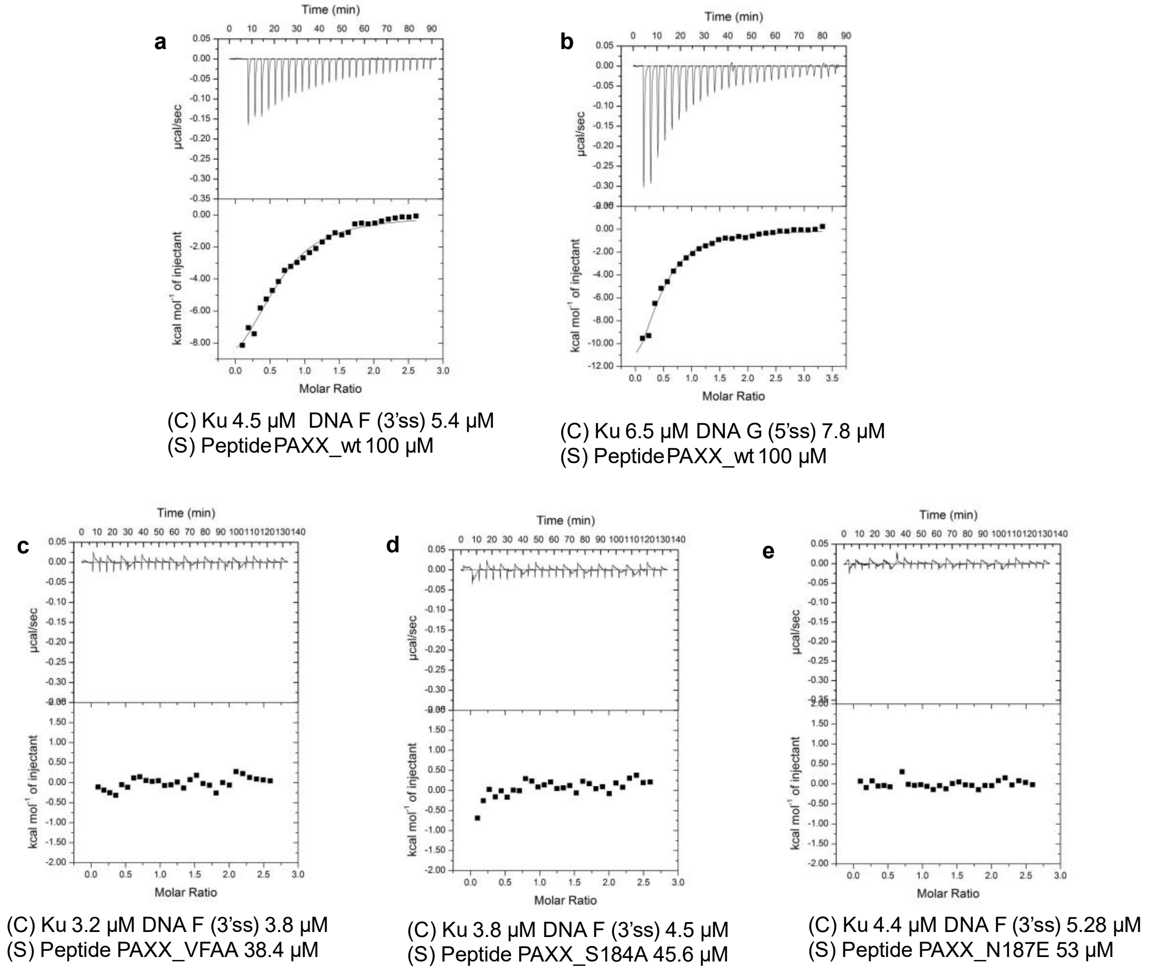

Fig. S6.

Isothermal titration calorimtery (ITC) of Ku70/80-DNA with the P-KBM of PAXX. a, b) Thermograms and isotherm obtained for Ku WT with P-KBM and DNA F or G. **c, d, e)** Thermograms and isotherm obtained for Ku WT with three PAXX mutants as labelled.

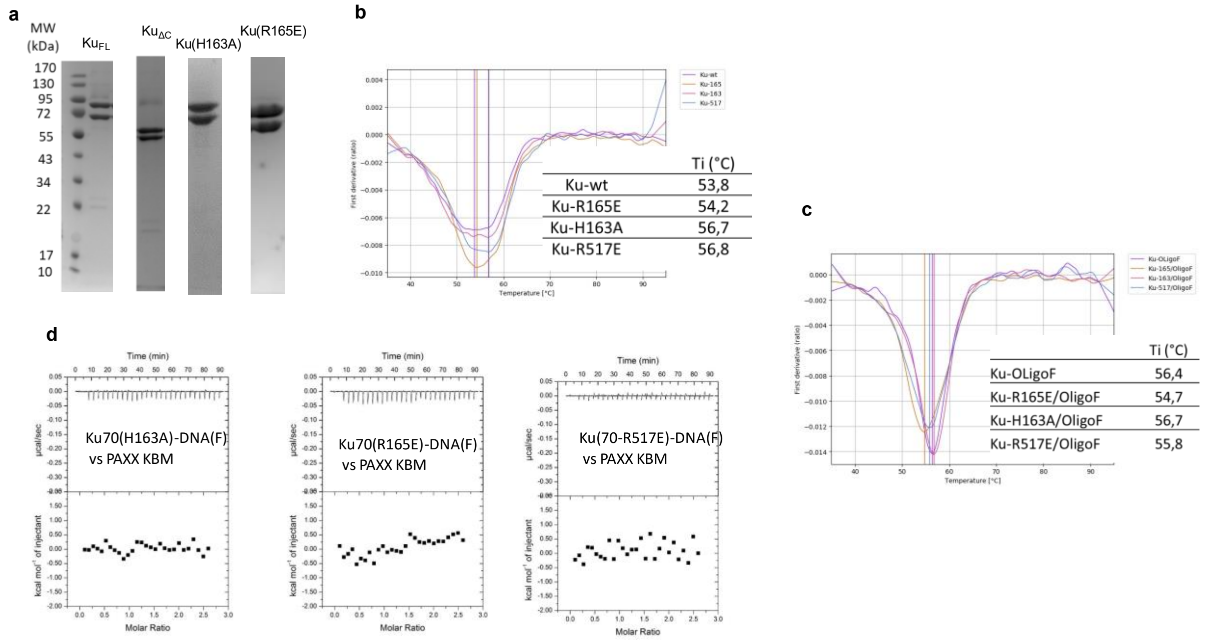

Fig. S7.

Ku70 variants with mutation in the PAXX binding site. a) SDS-PAGE of purified Ku_{FL}, $Ku_{\Delta C}$ and three variants of Ku_F with mutations H163A and R165E. **b)** nanoDSF analysis of Ku_{FL} wild type and three variants of in Ku_{FL} apo form **c)** nanoDSF analysis of Ku_{FL} wild type and three mutants in complex with DNA(F) **d)** calorimetry analysis (ITC) of Ku variants-DNA(F) complex versus PAXX KBM.

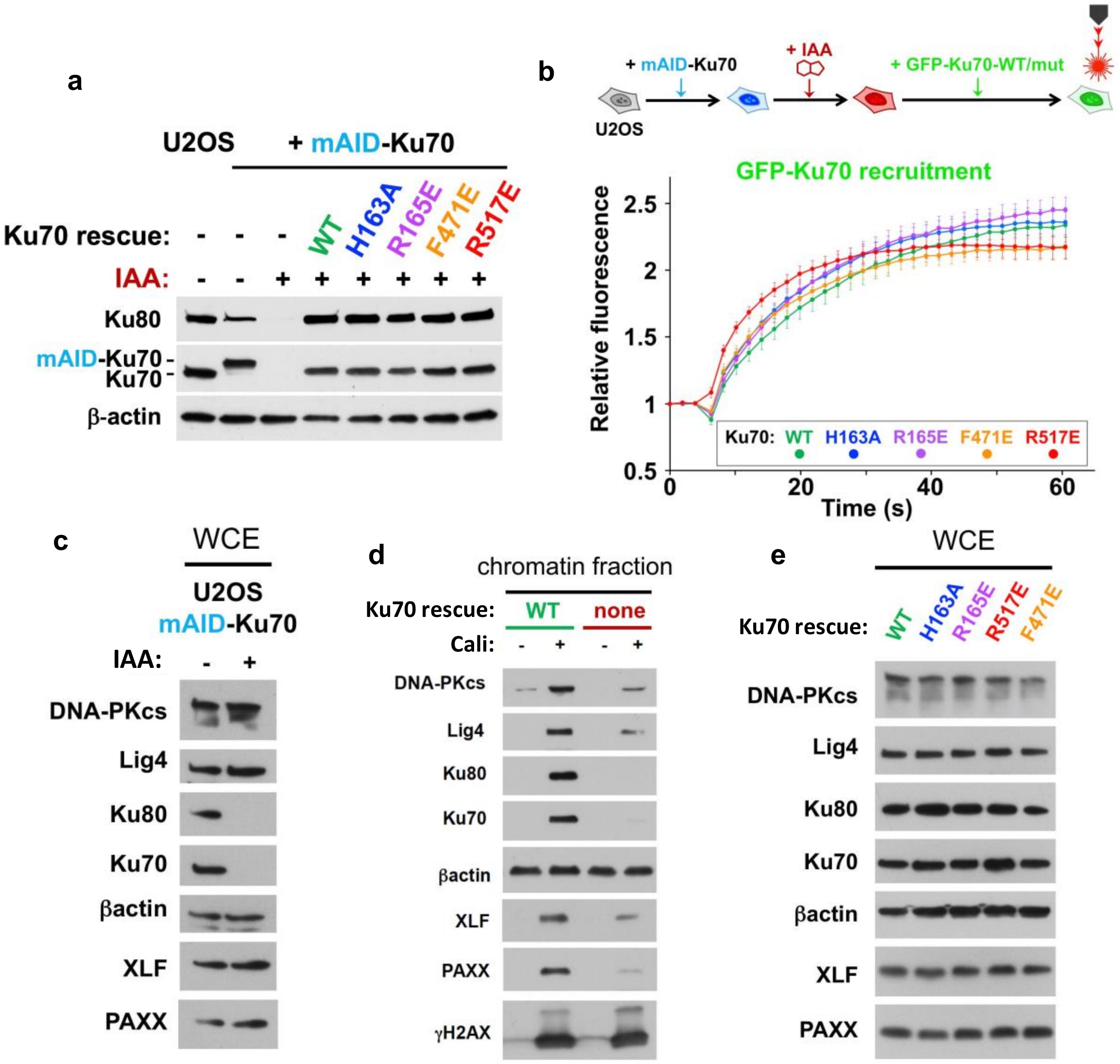

Fig. S8.

Expression of Ku70 mutants in U2OS cells. a) Control of expression of Ku70 constructs in U2OS cells**. b)** Monitoring of the recruitment of WT and mutant GFP-Ku70 at DNA damage sites over 1 min after laser micro-irradiation in U2OS cells. Results were plotted as mean values ± SEM. **c)** Control of expression of NHEJ protein in U2OS cells upon Ku extinction. **d)** Ku-dependency of NHEJ proteins recruitment to chromatin damaged with calicheamicin**. e)** Control of expression of NHEJ protein in Ku70 U2OS cells expressing Ku70 constructs**.**

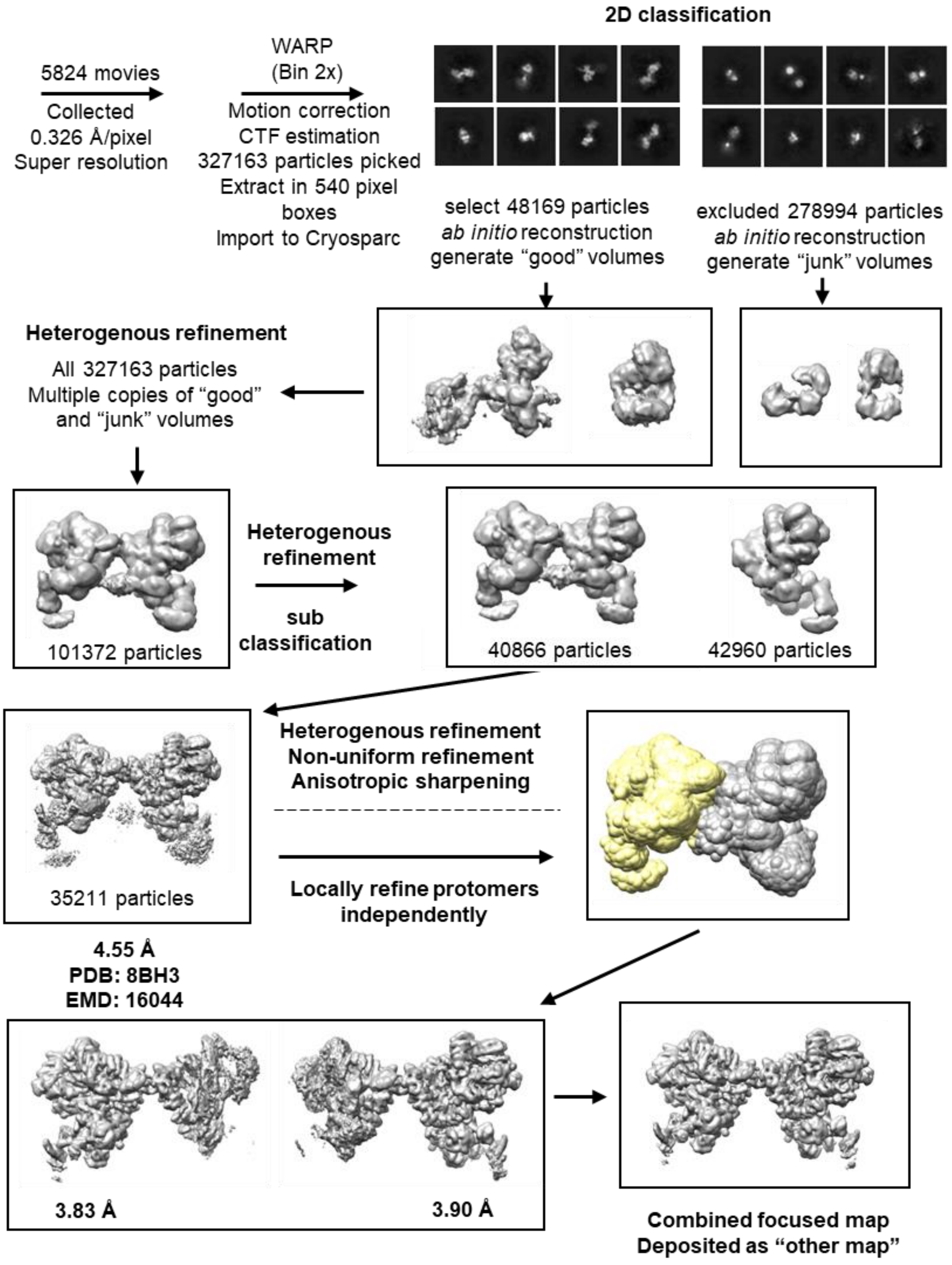

Fig. S9.

Single-particle cryo-EM image processing workflow for Ku80-mediated LR DNA-PK dimer with PAXX. Schematic showing particle picking using WARP and processing including 2D classification and *ab initio* reconstruction using CryoSPARC. The main class generated with the corresponding number of particles is shown and the final map after non-uniform refinement with a resolution for an FSC of 0.143 is given.

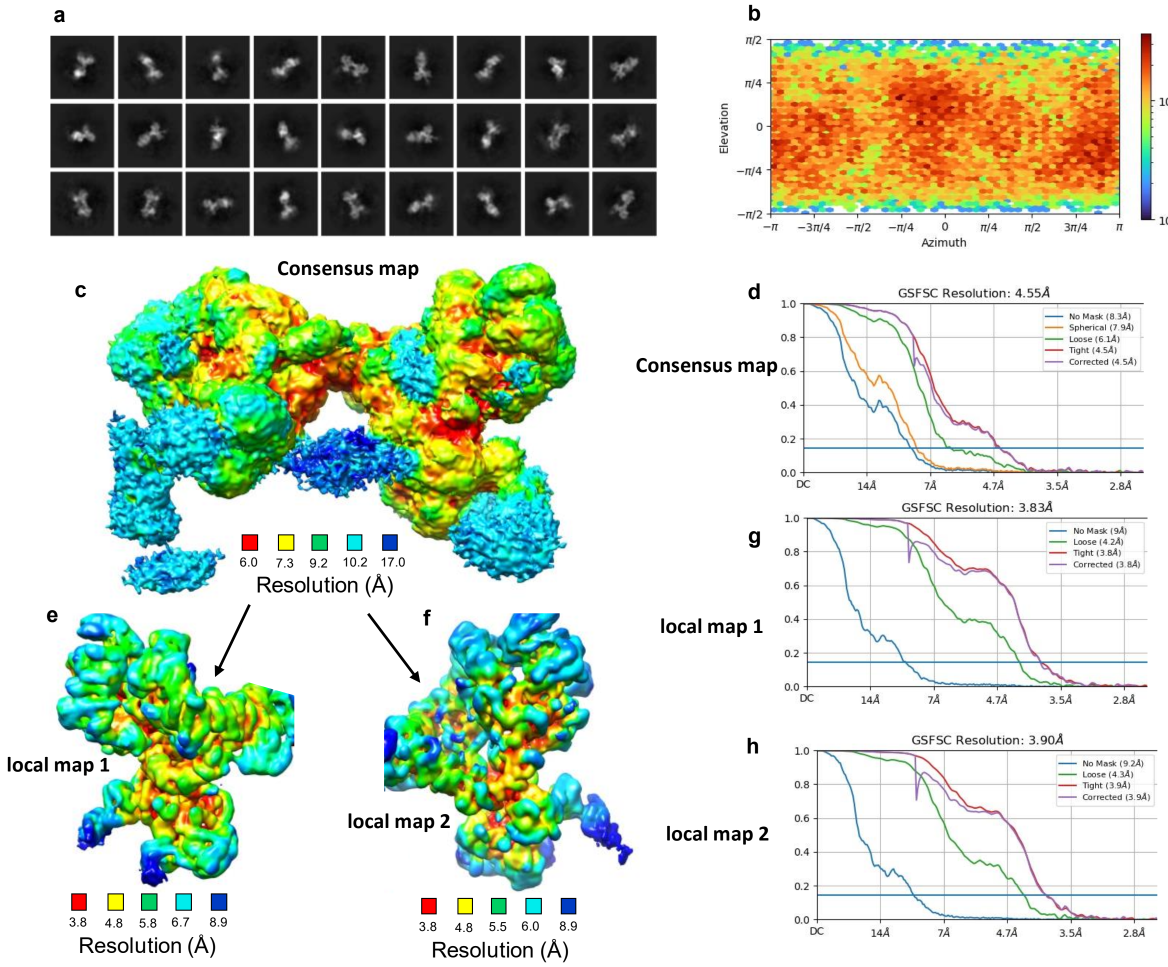

Fig. S10.

Cryo-EM data of Ku80-mediated LR DNA-PK dimer with PAXX. a) Examples of 2D classes. **b)** Angular distribution calculated in cryoSPARC for particle projections shown as a heat map. **c)** Local resolution map of the DNA-PK dimer with PAXX cryo-EM map. **d)** FSC resolution curves and viewing distribution plot for the consensus map. The colours corresponding to each resolution are displayed on the specific key chart below the maps. **e and f)** Local resolution of the two locally refined half maps. The colours corresponding to each resolution are displayed on the specific key chart below the maps. **g and h)** FSC resolution curves and viewing distribution plots of local maps 1 and 2.

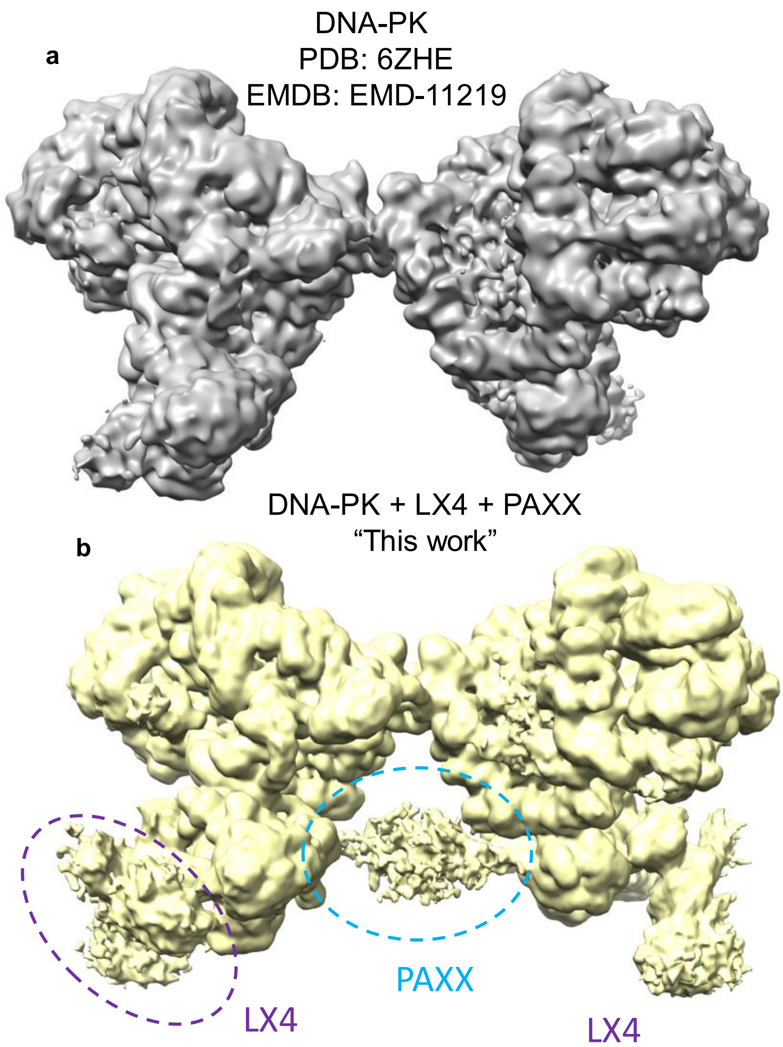

Fig. S11.
Cryo-EM map comparison of the Ku80-mediated dimer without (grey map) and with LX4 and PAXX (yellow map).

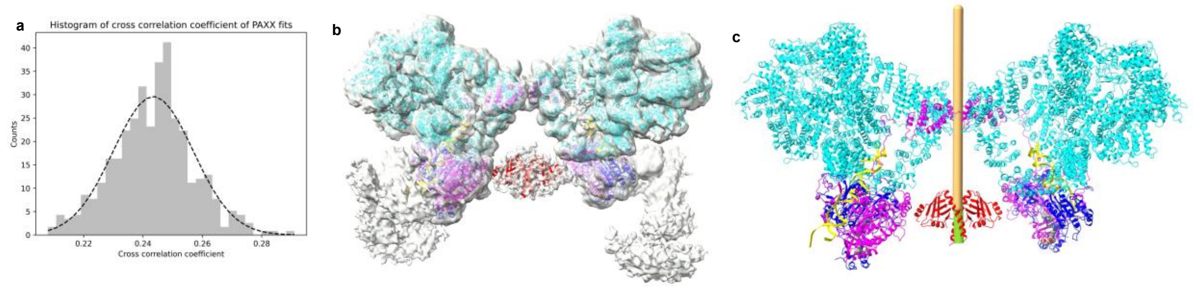

Fig. S12.

PAXX fitting in the Ku80-mediated DNA-PK dimer. a) The histogram of cross correlation coefficient score of the PAXX fits obtained using the ADP_EM exhaustive sampling procedure. **b)** The best fitting model of PAXX (shown as red cartoon) within the NHEJ super complex density map. The C-terminal peptide of PAXX bound to ku70/80 is shown as sphere model (coloured by atom type). **c)** The 2-fold axis of the PAXX predicted fit (shown as green cylinder) is shown in alignment with the 2-fold axis of DNA-PKcs (shown as light brown cylinder). This figure is same as Figure 2, but the density map corresponding the fits is not shown for clarity.

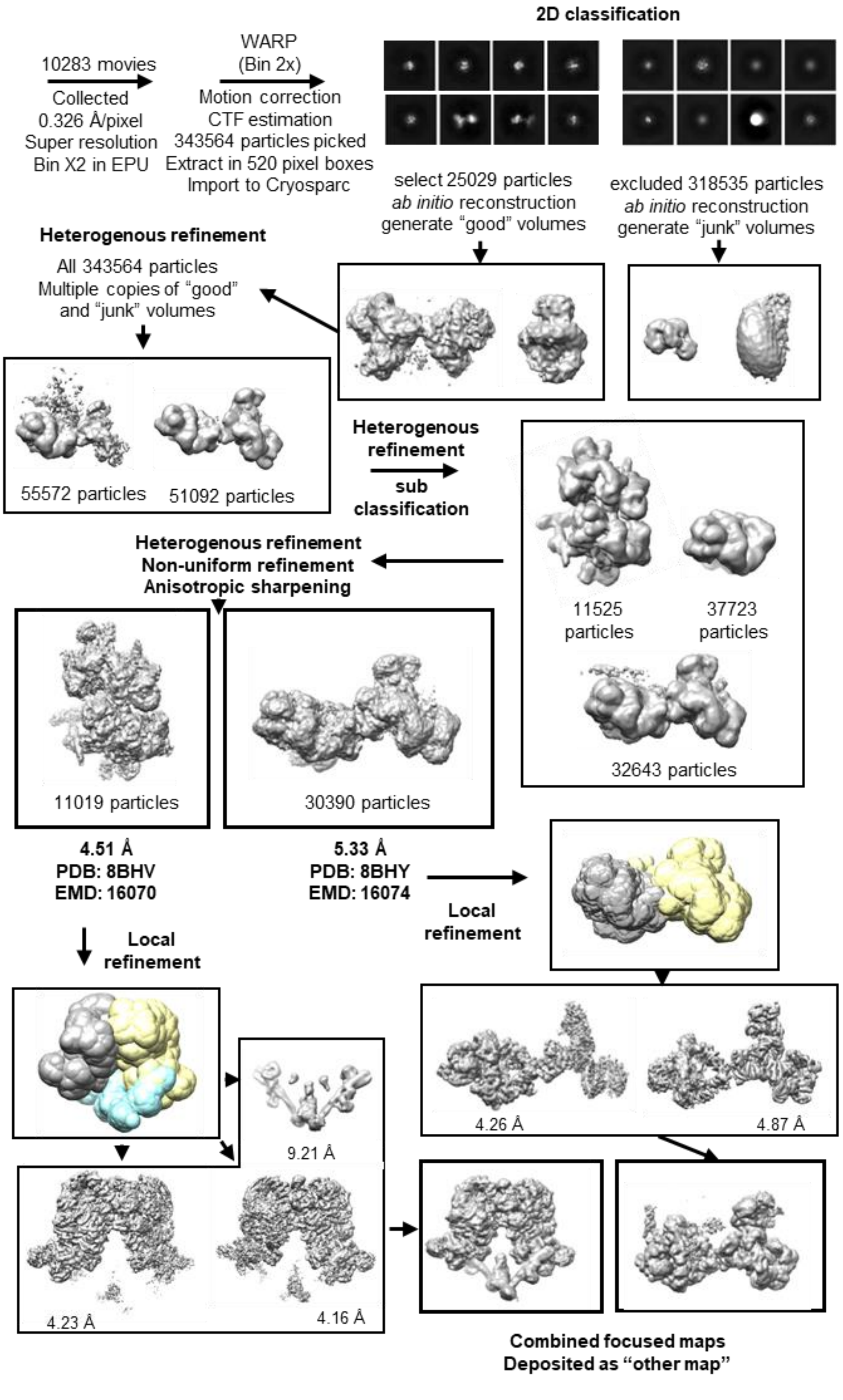

Fig. S13.

Single-particle cryo-EM image processing workflow for DNA-PK with LX4, XLF and PAXX. Schematic showing particle picking using WARP and processing including 2D classification and *ab initio* reconstruction using CryoSPARC. The two main classes generated with the corresponding number of particles is shown and the final two maps following non-uniform refinement with resolutions for an FSC of 0.143 are given.

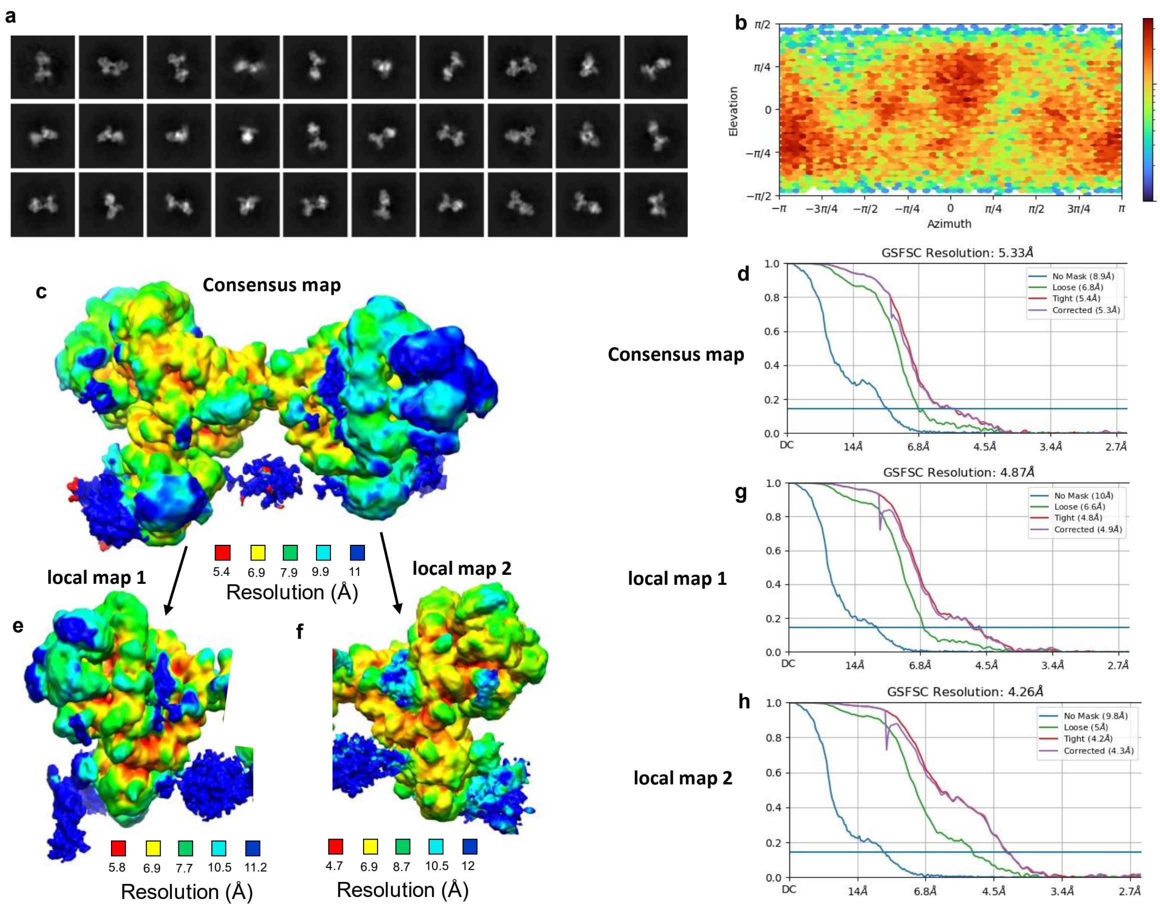

Fig. S14.

Cryo-EM data of Ku80-mediated LR DNA-PK dimer with PAXX and XLF. a) Examples of 2D classes. **b)** Angular distribution calculated in cryoSPARC for particle projections shown as a heat map. **c)** Local consensus resolution map of the DNA-PK dimer with PAXX and XLF cryo-EM map. The colours corresponding to each resolution are displayed on the specific key chart below the maps. **d)** FSC resolution curves and viewing distribution plot for the consensus map. **e and f)** Local resolution of the two locally refined half maps. The colours corresponding to each resolution are displayed on the specific key chart below the maps. **g and h)** FSC resolution curves and viewing distribution plot.

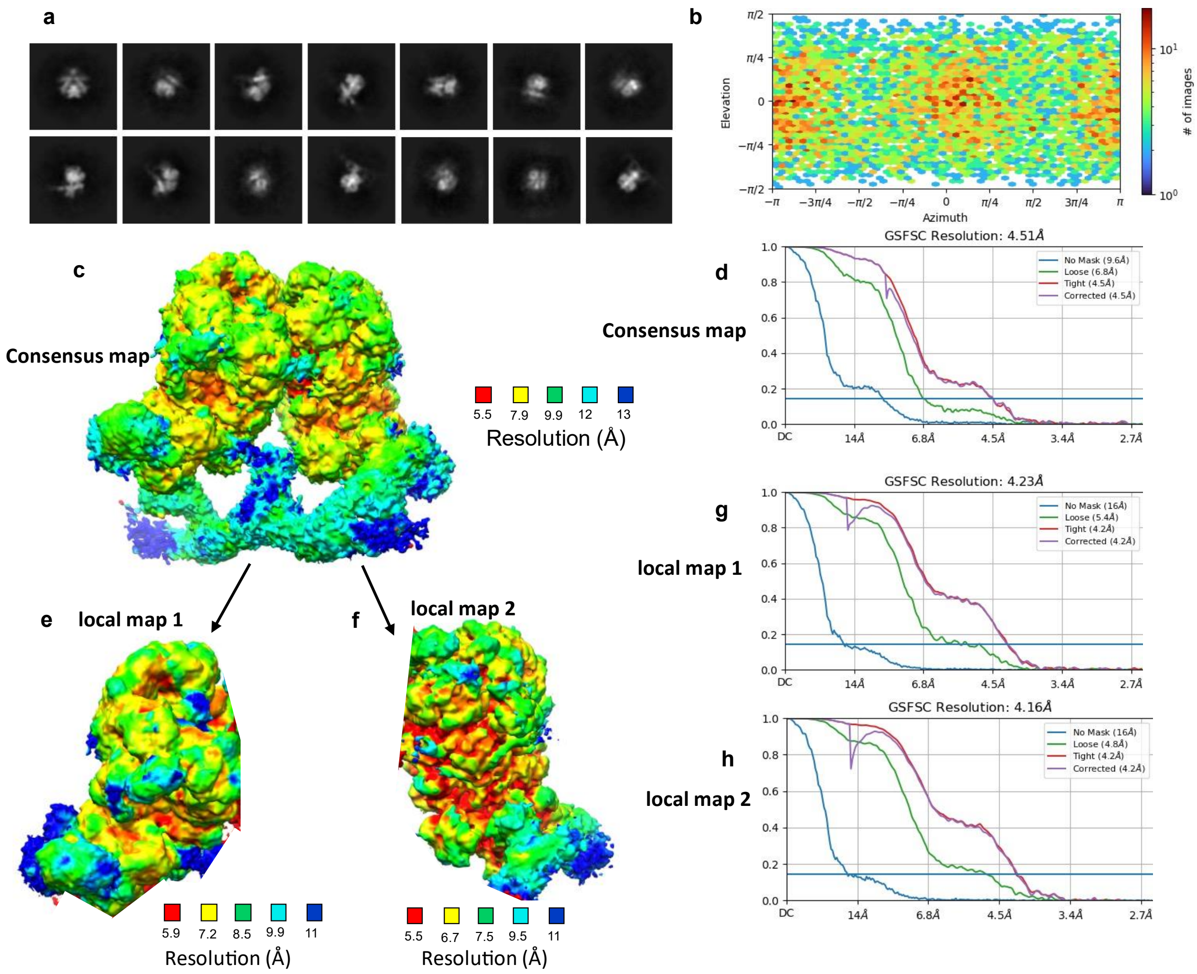

Fig. S15.

Cryo-EM data of XLF-mediated LR DNA-PK dimer with PAXX and XLF. a) Examples of 2D classes. **b)** Angular distribution calculated in cryoSPARC for particle projections shown as a heat map. **c)** Local resolution map of the DNA-PK dimer with PAXX and XLF consensus cryo-EM map. The colours corresponding to each resolution are displayed on the specific key chart below the maps. **d)** FSC resolution curves and viewing distribution plot of the consensus map. **e and f)** Local resolution of the two locally refined half maps. The colours corresponding to each resolution are displayed on the specific key chart below the maps. **g and h)** FSC resolution curves and viewing distribution plot of local map 1 and 2.

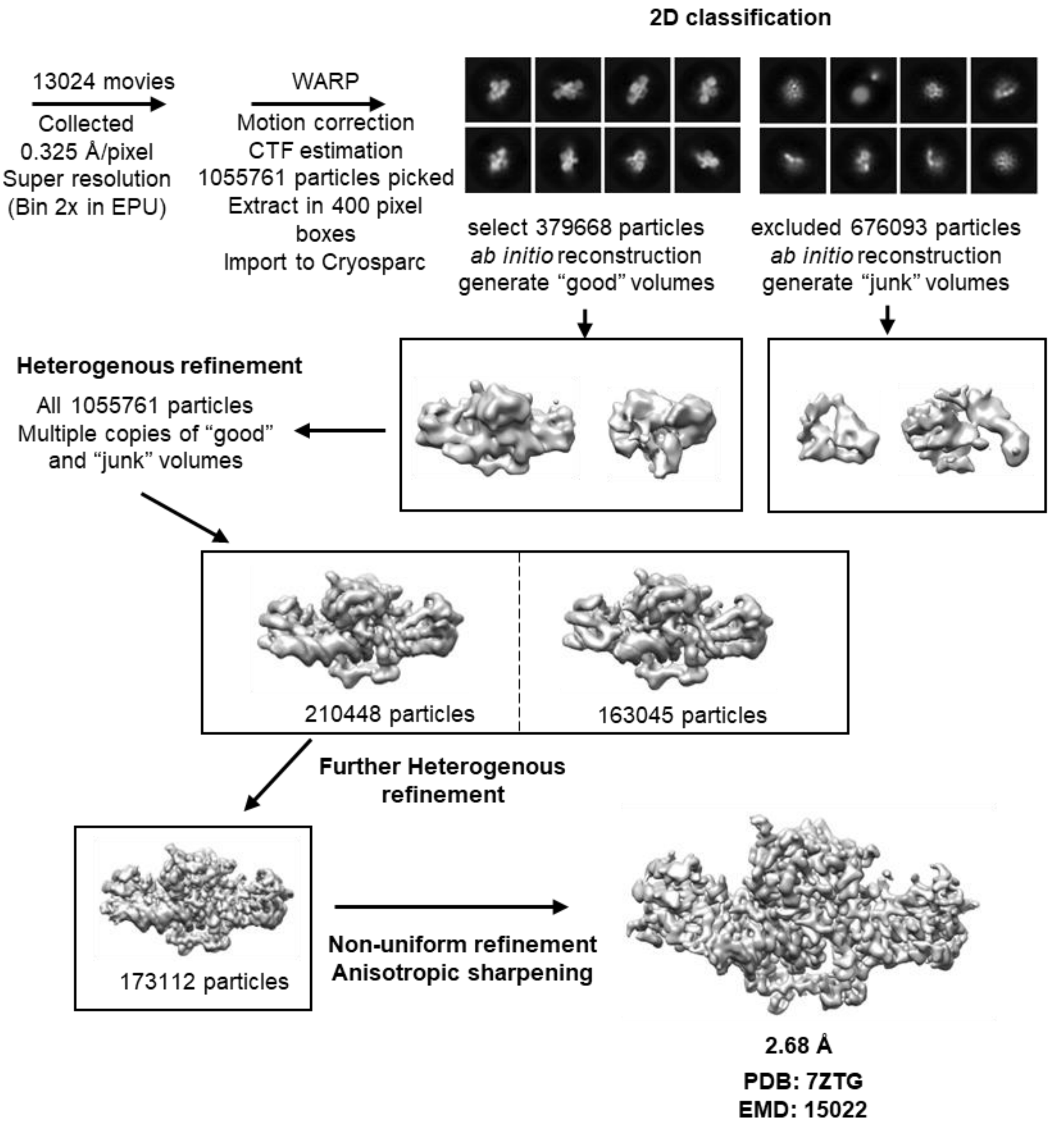

Fig. S16.

Single-particle cryo-EM image processing workflow for Ku70/80-DNA with PAXX and XLF. Schematic showing particle picking using WARP and processing including 2D classification and *ab initio* reconstruction using CryoSPARC. The main class generated with the corresponding number of particles is shown and the final map after non-uniform refinement with a resolution for an FSC of 0.143 is given.

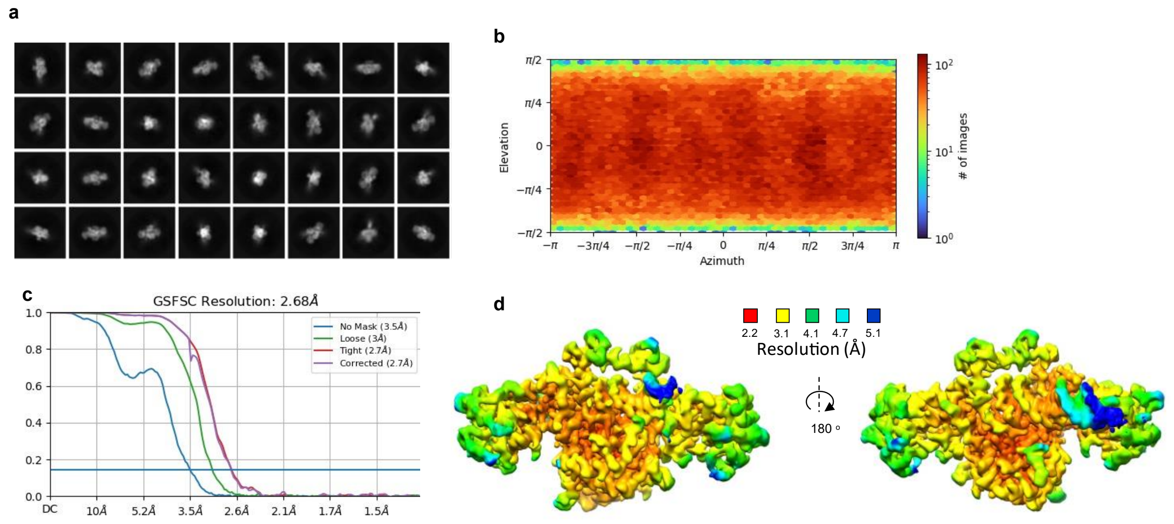

Fig. S17.

Cryo-EM data of Ku70/80-DNA with PAXX and XLF. a) Examples of 2D classes. **b)** Angular distribution calculated in cryoSPARC for particle projections shown as a heat map. **c)** FSC resolution curves and viewing distribution plot. **d)** Local resolution map of Ku70/80-DNA with PAXX and XLF cryo-EM map. The colours corresponding to each resolution are displayed on the specific key chart below the maps.

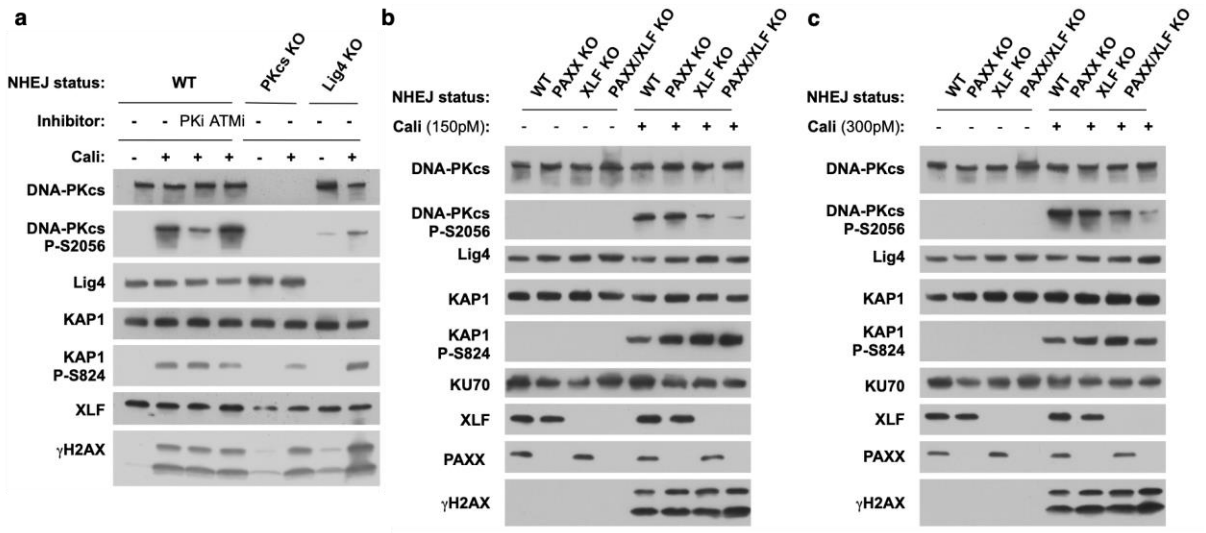

Fig. S18.

Assessing DSB synapsis via DNA-PKcs P-S2056. a) Western blotting on extracts from HEK293T cells as indicated, treated or not with 200 pM calicheamicin for 1 hr, after 1hr pre-incubation or not with an inhibitor of DNA-PK (NU7441, 3µM: PKi) or ATM (KU55933, 10µM:ATMi), as stated. **b-c)** Western blotting on extracts from HEK293T cells as indicated, treated or not with 150 pM **(b)** or 300 pM **(c)** calicheamicin for 1 hr ; **(b)** is the extended version of **Figure 5f**).

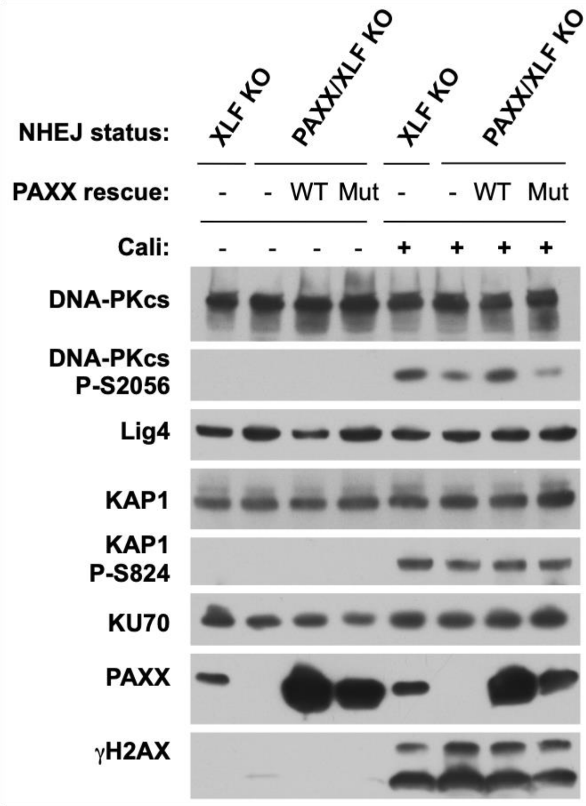

Fig. S19.

PAXX binding to Ku is necessary to maintain DNA ends synapsis in the absence of XLF. Western blotting on extracts from HEK293T cells as indicated, treated or not with 200 pM calicheamicin for 1 hr. PAXX/XLF double KO cells were complemented with either wild-type (WT) or mutated (F201A) PAXX as indicated.

	Ku-PAXX<br><br>PDB:7ZWA<br>EMD:14995	Ku-PAXX-XLF<br><br>PDB:7ZTG<br>EMD:15022	SC-PAXX<br>Ku80 dimer<br>PDB:8BH3<br>EMD:16044	SC-PAXX-XLF<br>Ku80 dimer<br>PDB :8BHY<br>EMD :16074	SC-PAXX-XLF<br>XLF dimer<br>PDB :8BHV<br>EMD :16070
**Data collection and processing**					
Detector	Gatan K3	Gatan K3	Gatan K3	Gatan K3	Gatan K3
Magnification	130k	130K	130K	130K	130K
Energy filter slit width (eV)	20	20	20	20	20
Voltage (kV)	300	300	300	300	300
Flux on detector (e/pix/sec)	13.689	17.82	12.55	15.24	15.24
Electron exposure on sample (e–/Å^2)	44.39	52.1	47.87	48.03	48.03
Target defocus range (µm)	0.8-2.4	0.8-2.4	0.8-2.5	0.9-2.4	0.9-2.4
Calibrated pixel size (Å)	0.65	0.65	1.304	1.304	1.304
Symmetry imposed	C1	C1	C1	C1	C1
Extraction box size (pixels)	380	400	540	520	520
Initial particle images (no.)	2143882	1055761	327163	343564	343564
Final particle images (no.)	161215	173112	35211	30390	11019
**Refinement**					
Map resolution at FSC=0.143 (Å)*	2.80	2.68	4.55	5.33	4.51
Model composition					
Non-hydrogen atoms	9038	8918	89330	90547	91487
Protein residues	1055	1039	11012	11026	11179
Nucleotides	31	30	106	102	103
B factor (Å^2)					
Protein	132.51	161.70	712.5	708.7	786.7
DNA	170.97	172.92	495.9	504.7	649.9
R.m.s deviations					
Bond lengths (Å)	0.002	0.002	0.003	0.003	0.004
Bond angles (°)	0.512	0.462	0.617	0.726	0.692
Validation					
Molprobity score	1.70	1.25	2.25	2.33	2.46
Clashscore	7.51	3.85	21.08	23.47	29.60
Poor rotamers (%)	0.00	0.00	0.04	0.12	0.05
Ramachandran plot					
Favored (%)	95.89	97.66	93.44	92.54	91.47
Allowed (%)	4.11	2.34	6.51	7.35	8.44
Disallowed (%)	0.00	0.00	0.06	0.11	0.09

Table S1. Cryo-EM data collection and refinement statistics. (Ku denotes Ku70/Ku80 heterodimer bound to DNA. SC denotes super complex of DNA-PK, XRCC4 and DNA Ligase 4).

	$Ku_{\Delta C}$ - pKBM PAXX- 3' overhang DNA PDB:8ASC
**Data collection**	
Space group	P1 21 1
Cell dimensions	
a, *b*, *c* (Å)	85.02, 428.57, 96.06
α, β, γ (°)	90.00, 90.01, 90.00
Resolution (Å)	2.973 (2.973) *
R_{sym} or R_{merge}	0.255 (1.661)
I / σI	6.0 (1.5)
Completeness (%)	86.7 (53.4)
Redundancy	7.8 (6.6)
**Refinement**	
Resolution (Å)	45.32 – 2.95
No. reflections	34 164
R_{work} / R_{free}	0.252/0.298
No. atoms	
Protein	33 555
Ligand/ion	2 808
Water	25
B-factors	
Protein	97.31
DNA/ion	146.77/87.76
Water	28.41
R.m.s. deviations	
Bond lengths (Å)	0.0059
Bond angles (°)	1.248

Table S2.

Data collection and refinement statistics for the X-ray crystallography structure of Ku70/80-DNA-PAXX PKBM.

* Data set for one crystal. *Values in parentheses are for highest-resolution shell.
[AU: Equations defining various *R*-values are standard and hence are no longer defined in the footnotes.]
[AU: Ramachandran statistics should be in Methods section at the end of Refinement subsection.]
[AU: Wavelength of data collection, temperature and beamline should all be in Methods section.]

Movie S1.

Movie to show the transition between Ku70/80 without PAXX and with PAXX bound.

REFERENCES AND NOTES

1. B. Zhao, E. Rothenberg, D. A. Ramsden, M. R. Lieber, The molecular basis and disease relevance of non-homologous DNA end joining. *Nat. Rev. Mol. Cell Biol.* **21**, 765–781 (2020).

2. B. M. Stinson, J. J. Loparo, Repair of DNA double-strand breaks by the nonhomologous end joining pathway. *Annu. Rev. Biochem.* **90**, 137–164 (2021).

3. D. Ghosh, S. C. Raghavan, Nonhomologous end joining: New accessory factors fine tune the machinery. *Trends Genet.* **37**, 582–599 (2021).

4. K. Meek, V. Dang, S. P. Lees-Miller, DNA-PK: The means to justify the ends? *Adv. Immunol.* **99**, 33–58 (2008).

5. N. Jette, S. P. Lees-Miller, The DNA-dependent protein kinase: A multifunctional protein kinase with roles in DNA double strand break repair and mitosis. *Prog. Biophys. Mol. Biol.* **117**, 194–205 (2015).

6. A. K. Chaplin, S. W. Hardwick, S. Liang, A. K. Stavridi, A. Hnizda, L. R. Cooper, T. M. De Oliveira, D. Y. Chirgadze, T. L. Blundell, Dimers of DNA-PK create a stage for DNA double-strand break repair. *Nat. Struct. Mol. Biol.* **28**, 13–19 (2020).

7. A. K. Chaplin, S. W. Hardwick, A. K. Stavridi, C. J. Buehl, N. J. Goff, V. Ropars, S. Liang, T. M. de Oliveira, D. Y. Chirgadze, K. Meek, J.-B. Charbonnier, T. L. Blundell, Cryo-EM of NHEJ supercomplexes provides insights into DNA repair. *Mol. Cell* **81**, 3400–3409.e3 (2021).

8. S. Chen, L. Lee, T. Naila, S. Fishbain, A. Wang, A. E. Tomkinson, S. P. Lees-Miller, Y. He, Structural basis of long-range to short-range synaptic transition in NHEJ. *Nature* **593**, 294–298 (2021).

9. T. G. W. Graham, J. C. Walter, J. J. Loparo, Two-stage synapsis of DNA ends during non-homologous end joining. *Mol. Cell* **61**, 850–858 (2016).

10. C. Nemoz, V. Ropars, P. Frit, A. Gontier, P. Drevet, J. Yu, R. Guerois, A. Pitois, A. Comte, C. Delteil, N. Barboule, P. Legrand, S. Baconnais, Y. Yin, S. Tadi, E. Barbet-Massin, I. Berger, E. le Cam, M. Modesti, E. Rothenberg, P. Calsou, J. B. Charbonnier, XLF and APLF bind Ku80 at two remote sites to ensure DNA repair by non-homologous end joining. *Nat. Struct. Mol. Biol.* **25**, 971–980 (2018).

11. J. Tang, Z. Li, Q. Wu, M. Irfan, W. Li, X. Liu, Role of paralogue of XRCC4 and XLF in DNA damage repair and cancer development. *Front. Immunol.* **13**, 852453 (2022).

12. P. Frit, V. Ropars, M. Modesti, J. B. Charbonnier, P. Calsou, Plugged into the Ku-DNA hub: The NHEJ network. *Prog. Biophys. Mol. Biol.* **147**, 62–76 (2019).

13. T. Ochi, A. N. Blackford, J. Coates, S. Jhujh, S. Mehmood, N. Tamura, J. Travers, Q. Wu, V. M. Draviam, C. V. Robinson, T. L. Blundell, S. P. Jackson, DNA repair. PAXX, a paralog of XRCC4 and XLF, interacts with Ku to promote DNA double-strand break repair. *Science* **347**, 185–188 (2015).

14. S. K. Tadi, C. Tellier-Lebègue, C. Nemoz, P. Drevet, S. Audebert, S. Roy, K. Meek, J.-B. Charbonnier, M. Modesti, PAXX is an accessory c-NHEJ factor that associates with Ku70 and has overlapping functions with XLF. *Cell Rep.* **17**, 541–555 (2016).

15. J. L. Wang, C. Duboc, Q. Wu, T. Ochi, S. Liang, S. E. Tsutakawa, S. P. Lees-Miller, M. Nadal, J. A. Tainer, T. L. Blundell, T. R. Strick, Dissection of DNA double-strand-break repair using novel single-molecule forceps. *Nat. Struct. Mol. Biol.* **25**, 482–487 (2018).

16. A. Craxton, D. Munnur, R. Jukes-Jones, G. Skalka, C. Langlais, K. Cain, M. Malewicz, PAXX and its paralogs synergistically direct DNA polymerase λ activity in DNA repair. *Nat. Commun.* **9**, 3877 (2018).

17. A. Dewan, M. Xing, M. B. Lundbaek, R. Gago-Fuentes, C. Beck, P. A. Aas, N.-B. Liabakk, S. Saeterstad, K. T. P. Chau, B. M. Kavli, V. Oksenych, Robust DNA repair in PAXX-deficient mammalian cells. *FEBS Open Bio* **8**, 442–448 (2018).

18. V. Kumar, F. W. Alt, R. L. Frock, PAXX and XLF DNA repair factors are functionally redundant in joining DNA breaks in a G1-arrested progenitor B-cell line. *Proc. Natl. Acad. Sci. U.S.A.* **113**, 10619–10624 (2016).

19. C. Lescale, H. Lenden Hasse, A. N. Blackford, G. Balmus, J. J. Bianchi, W. Yu, L. Bacoccina, A. Jarade, C. Clouin, R. Sivapalan, B. Reina-San-Martin, S. P. Jackson, L. Deriano, Specific roles of XRCC4 paralogs PAXX and XLF during V(D)J recombination. *Cell Rep.* **16**, 2967–2979 (2016).

20. M. Xing, M. Yang, W. Huo, F. Feng, L. Wei, W. Jiang, S. Ning, Z. Yan, W. Li, Q. Wang, M. Hou, C. Dong, R. Guo, G. Gao, J. Ji, S. Zha, L. Lan, H. Liang, D. Xu, Interactome analysis identifies a new paralogue of XRCC4 in non-homologous end joining DNA repair pathway. *Nat. Commun.* **6**, 6233 (2015).

21. V. Abramowski, O. Etienne, R. Elsaid, J. Yang, A. Berland, L. Kermasson, B. Roch, S. Musilli, J.-P. Moussu, K. Lipson-Ruffert, P. Revy, A. Cumano, F. D. Boussin, J.-P. de Villartay, PAXX and Xlf interplay revealed by impaired CNS development and immunodeficiency of double KO mice. *Cell Death Differ.* **25**, 444–452 (2018).

22. G. Balmus, A. C. Barros, P. W. G. Wijnhoven, C. Lescale, H. L. Hasse, K. Boroviak, C. le Sage, B. Doe, A. O. Speak, A. Galli, M. Jacobsen, L. Deriano, D. J. Adams, A. N. Blackford, S. P. Jackson, Synthetic lethality between PAXX and XLF in mammalian development. *Genes Dev.* **30**, 2152–2157 (2016).

23. R. Gago-Fuentes, M. Xing, S. Saeterstad, A. Sarno, A. Dewan, C. Beck, S. Bradamante, M. Bjørås, V. Oksenych, Normal development of mice lacking PAXX, the paralogue of XRCC4 and XLF. *FEBS Open Bio* **8**, 426–434 (2018).

24. X. Liu, Z. Shao, W. Jiang, B. J. Lee, S. Zha, PAXX promotes KU accumulation at DNA breaks and is essential for end-joining in XLF-deficient mice. *Nat. Commun.* **8**, 13816 (2017).

25. P. J. Hung, B.-R. Chen, R. George, C. Liberman, A. J. Morales, P. Colon-Ortiz, J. K. Tyler, B. P. Sleckman, A. L. Bredemeyer, Deficiency of XLF and PAXX prevents DNA double-

strand break repair by non-homologous end joining in lymphocytes. *Cell Cycle* **16**, 286–295 (2017).

26. S. Roy, A. J. de Melo, Y. Xu, S. K. Tadi, A. Négrel, E. Hendrickson, M. Modesti, K. Meek, XRCC4/XLF interaction is variably required for DNA repair and is not required for ligase IV stimulation. *Mol. Cell. Biol.* **35**, 3017–3028 (2015).

27. M. Arora, S. Kumari, J. Singh, A. Chopra, S. S. Chauhan, PAXX, not NHEJ1 is an independent prognosticator in colon cancer. *Front. Mol. Biosci.* **7**, 584053 (2020).

28. T. Natsume, T. Kiyomitsu, Y. Saga, M. T. Kanemaki, Rapid protein depletion in human cells by auxin-inducible degron tagging with short homology donors. *Cell Rep.* **15**, 210–218 (2016).

29. J. Drouet, C. Delteil, J. Lefrançois, P. Concannon, B. Salles, P. Calsou, DNA-dependent protein kinase and XRCC4-DNA ligase IV mobilization in the cell in response to DNA double strand breaks. *J. Biol. Chem.* **280**, 7060–7069 (2005).

30. J. I. Garzon, J. Kovacs, R. Abagyan, P. Chacon, ADP_EM: Fast exhaustive multi-resolution docking for high-throughput coverage. *Bioinformatics* **23**, 427–433 (2007).

31. E. Weterings, N. S. Verkaik, H. T. Bruggenwirth, J. H. Hoeijmakers, D. C. van Gent, The role of DNA dependent protein kinase in synapsis of DNA ends. *Nucleic Acids Res.* **31**, 7238–7246 (2003).

32. K. Meek, P. Douglas, X. Cui, Q. Ding, S. P. Lees-Miller, Trans autophosphorylation at DNA-dependent protein kinase's two major autophosphorylation site clusters facilitates end processing but not end joining. *Mol. Cell. Biol.* **27**, 3881–3890 (2007).

33. J. Cottarel, P. Frit, O. Bombarde, B. Salles, A. Négrel, S. Bernard, P. A. Jeggo, M. R. Lieber, M. Modesti, P. Calsou, A noncatalytic function of the ligation complex during nonhomologous end joining. *J. Cell Biol.* **200**, 173–186 (2013).

34. V. Menon, L. F. Povirk, XLF/Cernunnos: An important but puzzling participant in the nonhomologous end joining DNA repair pathway. *DNA Repair (Amst)* **58**, 29–37 (2017).

35. H. Sharif, Y. Li, Y. Dong, L. Dong, W. L. Wang, Y. Mao, H. Wu, Cryo-EM structure of the DNA-PK holoenzyme. *Proc. Natl. Acad. Sci. U.S.A.* **114**, 7367–7372 (2017).

36. E. F. Pettersen, T. D. Goddard, C. C. Huang, G. S. Couch, D. M. Greenblatt, E. C. Meng, T. E. Ferrin, UCSF chimera—A visualization system for exploratory research and analysis. *J. Comput. Chem.* **25**, 1605–1612 (2004).

37. P. Emsley, B. Lohkamp, W. G. Scott, K. Cowtan, Features and development of Coot. *Acta Crystallogr. D Biol. Crystallogr.* **66**, 486–501 (2010).

38. E. F. Pettersen, T. D. Goddard, C. C. Huang, E. C. Meng, G. S. Couch, T. I. Croll, J. H. Morris, T. E. Ferrin, UCSF ChimeraX: Structure visualization for researchers, educators, and developers. *Protein Sci.* **30**, 70–82 (2021).

39. W. Kabsch, XDS. *Acta Crystallogr. D Biol. Crystallogr.* **66**, 125–132 (2010).

40. M. D. Winn, C. C. Ballard, K. D. Cowtan, E. J. Dodson, P. Emsley, P. R. Evans, R. M. Keegan, E. B. Krissinel, A. G. W. Leslie, A. McCoy, S. J. McNicholas, G. N. Murshudov, N. S. Pannu, E. A. Potterton, H. R. Powell, R. J. Read, A. Vagin, K. S. Wilson, Overview of the CCP4 suite and current developments. *Acta Crystallogr. D Biol. Crystallogr.* **67**, 235–242 (2011).

41. C. Vonrhein, C. Flensburg, P. Keller, A. Sharff, O. Smart, W. Paciorek, T. Womack, G. Bricogne, Data processing and analysis with the autoPROC toolbox. *Acta Crystallogr. D Biol. Crystallogr.* **67**, 293–302 (2011).

42. J. R. Walker, R. A. Corpina, J. Goldberg, Structure of the Ku heterodimer bound to DNA and its implications for double-strand break repair. *Nature* **412**, 607–614 (2001).

43. Q. Cheng, N. Barboule, P. Frit, D. Gomez, O. Bombarde, B. Couderc, G.-S. Ren, B. Salles, P. Calsou, Ku counteracts mobilization of PARP1 and MRN in chromatin damaged with DNA double-strand breaks. *Nucleic Acids Res.* **39**, 9605–9619 (2011).

44. C. Fu, W. P. Donovan, O. Shikapwashya-Hasser, X. Ye, R. H. Cole, Hot fusion: An efficient method to clone multiple DNA fragments as well as inverted repeats without ligase. *PLOS ONE* **9**, e115318 (2014).

45. D. S. F. Biard, Untangling the relationships between DNA repair pathways by silencing more than 20 DNA repair genes in human stable clones. *Nucleic Acids Res.* **35**, 3535–3550 (2007).

46. G. E. Crooks, G. Hon, J. M. Chandonia, S. E. Brenner, WebLogo: A sequence logo generator. *Genome Res.* **14**, 1188–1190 (2004).
